# Supplementary material for: Ultra-rapid and highly efficient enrichment of organic pollutants via magnetic mesoporous nanosponge for ultrasensitive nanosensors
Source: Nat Commun. 2021 Nov 25;12:6849. doi: 10.1038/s41467-021-27100-2 (PMC8617178; doi:10.1038/s41467-021-27100-2)
Supplement: Supplementary file 1 — Supplementary Information [file 41467_2021_27100_MOESM1_ESM.docx]

Supplementary Information

**Ultra-rapid and highly efficient enrichment of organic** **pollutants via magnetic nanoparticles/mesoporous nanosponge compounds for** **complex matrix ultrasensitive nanosensors**

Lingling Zhang^1,#^, Yu Guo^1,#^, Rui Hao^1^, Yafei Shi^1^, Hongjun You^2^, Hu Nan^3^, Yanzhu Dai^3^, Danjun Liu^4^, Dangyuan Lei^4^, Jixiang Fang^1,2,*^

^1^School of Electronic Science and Engineering, Faculty of Electronic and Information Engineering, Xi’an Jiaotong University, Xi’an, Shaanxi, 710049, China

^2^Key Laboratory of Biomedical Information Engineering of Ministry of Education, School of Life Science and Technology, Xi’an Jiaotong University, Xi’an, Shaanxi, 710049, China

^3^School of Microelectronics, Faculty of Electronic and Information Engineering, Xi’an Jiaotong University, Xi’an Jiaotong University, Xi’an, Shaanxi, 710049, China

^4^Department of Materials Science and Engineering, City University of Hong Kong, 83 Tat Chee Avenue, Kowloon, Hong Kong, 999077, China

^#^These authors contributed equally: Lingling Zhang, Yu Guo

*To whom correspondence may be addressed. E-mail: [jxfang@mail.xjtu.edu.cn](mailto:jxfang@mail.xjtu.edu.cn),

**The Supporting Information includes:**

Part S1: Materials, Preparation of MNEPI-CDP and Characterization

Part S2: Supplementary Figure 1 to Supplementary Figure 39, Supplementary Table 1 to Supplementary Table 6

**Materials.** β-cyclodextrin (β-CD, ≥98.0%) and tetrafluoroterephthalonitrile (TFT, ≥99.0%)，Tetrahydrofuran (THF, anhydrous, ≥99.9%), N,N-Dimethylformamide (DMF, anhydrous, ≥99.8%), potassium carbonate (K_2_CO_3_, anhydrous, ≥99.5%), gold chloride trihydrate (HAuCl_4_·3H_2_O, ≥99.9%), Bisphenol A (BPA, ≥99.0%), Bisphenol S (BPS, ≥99.0%), 1-naphthyl amine (1-NA, ≥99.0%), 2-naphthol (2-NO, ≥99.5%), carbendazim (≥99.0%), epichlorohydrin (EPI, ≥99.5%), tetramethyl thiuram disulfide (TMTD, 97%), N,N-Dimethylformamide (DMF, ≥99.9%), pentadecafluorooctanoic acid (PFOA, 98%), hexadecyl trimethyl ammonium bromide (CTAB, ≥99%), chlormequat chloride (CCC, 98%), hexamethylenetetramine (HMTA, ≥99.0%), diquat (analytical standard), anthracene (≥99.0%), malachite green (MG, 95%) and sunset yellow (SY, ≥95%) were obtained from Shanghai Aladdin Biochemical Technology Co., Ltd and used without further purification. Parathion (≥99.0%) was bought from J&K Scientific Ltd. Iron chloride hexahydrate (FeCl_3_·6H_2_O, ≥99.0%), trisodium citrate (C_6_H_5_Na_3_O_7_, ≥99.5%), ethylene glycol (EG, (CH_2_OH)_2_, ≥99.5%), sodium acetate trihydrate (CH_3_COONa, ≥99.0%), ethanol (CH_3_CH_2_OH, ≥95.0%), dichloromethane (CH_2_Cl_2_, ≥99.5%) and hydrochloric acid (HCl, 95%) were purchased from Sinopharm Chemical Reagent Co., Ltd. Norit ROW 0.8 supra extruded activated carbon (NAC) pellets were purchased from Sigma Aldrich and ground into a fine powder before use. Industrial wastewater, pond water and soil water were got from Engineering Workshop, Flying Tower Fountain and East Garden in Xi'an Jiaotong University, respectively. Φ13 mm needle cartridge poly tetra fluoroethylene (PTFE) membrane filter with bore diameter of 0.22 µm was purchased from Tianjin Jinteng Experiment Equipment Co. Ltd. Teflon membrane (polytetrafluoroethylene, PTFE) with 0.1μm pore size, 70 μm thickness and glass microfiber filter (Whatman no.1820-047) were purchased from Whatman Corporation. Perfluorinated fluids (Krytox, GPL105) were purchased from Dupont Corporation. Millipore ultrapure water (≥18 MΩ) was used in all experiments.

**Preparation of** **magnetic nanoparticles immobilized β-CD polymer using EPI as cross-linking agent (****MNEPI-CDP).** β-CD (1.20 g, 10.56 mmol) and dried Fe_3_O_4_ (0.06 g) were dissolved in aqueous NaOH (6.25 N, 20.0 mL) at 60 °C. Epichlorohydrin (10.0 mL, 129 mmol,) was added to this solution dropwise while stirring vigorously at 60 °C. The mixture turned into a brown gel within 1 h, after which 40 mL of deionized H_2_O was added, and the mixture was magnetically separated by magnet. The solid was washed by soaking in deionized H_2_O (4 x 150 mL) for 15 min, THF (3 x 50 mL) for 30 min and CH_2_Cl_2_ (1 x 15 mL) for 15 min. The solid was finally dried under high vacuum for 2 d at rt to give MNEPI-CDP.

**SERS measurement by the solution-based aggregation approach.** SERS spectra were obtained by mixing 2 mL target molecules in ethanol, 100 µL NaCl (1 M), and 500 µL Au NPs in cuvette.

**Characterization.** The morphology of the product was characterized via transmission electron microscope (TEM, JEOL JEM-2100 with an accelerating voltage of 200 kV). The UV-vis spectra were recorded via ultraviolet-visible spectroscopy (Agilent, Carry 60). N_2_ adsorption, desorption isotherms and surface area measurements were conducted on an ASAP 2020 Plus HD88. Infrared spectroscopy (FT-IR) was obtained by Micro-Infrared Spectroscopy (MIR, Bruker VERTEX70). The fluorescence spectra in this study were directly in solution by fluorescence spectrophotometer (Horiba PL spectrometer, Fluorolog-3), with 280 nm excitation wavelength, 5 nm slit width and 450W power xenon lamp. SERS signals were measured using a portable Raman spectrometer (BWTEK, i-Raman), with a 785 nm laser, using 40× objective, 20 s integration time, and 5% laser power (15 mW).

a


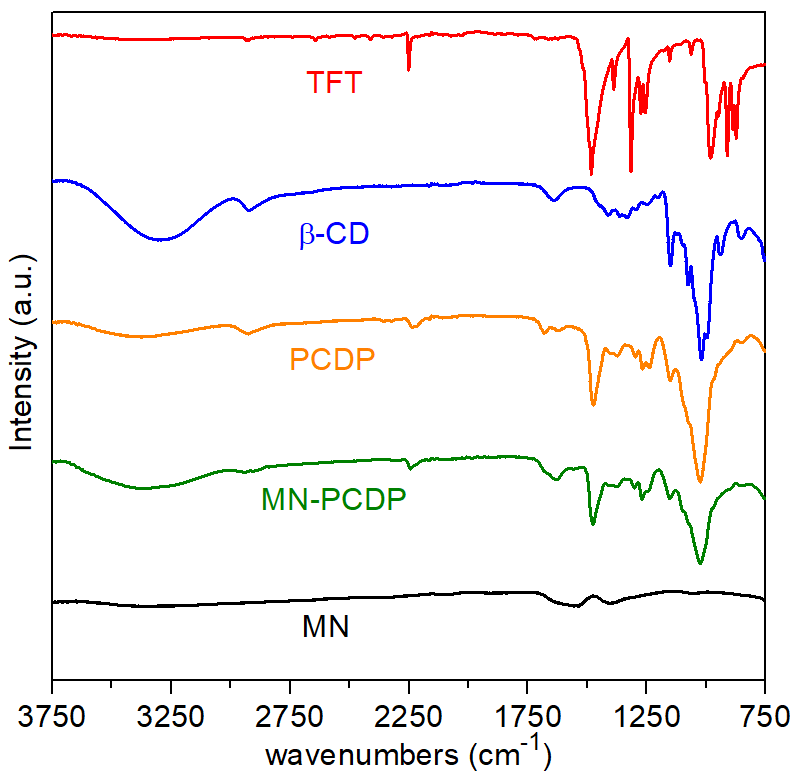


d

b


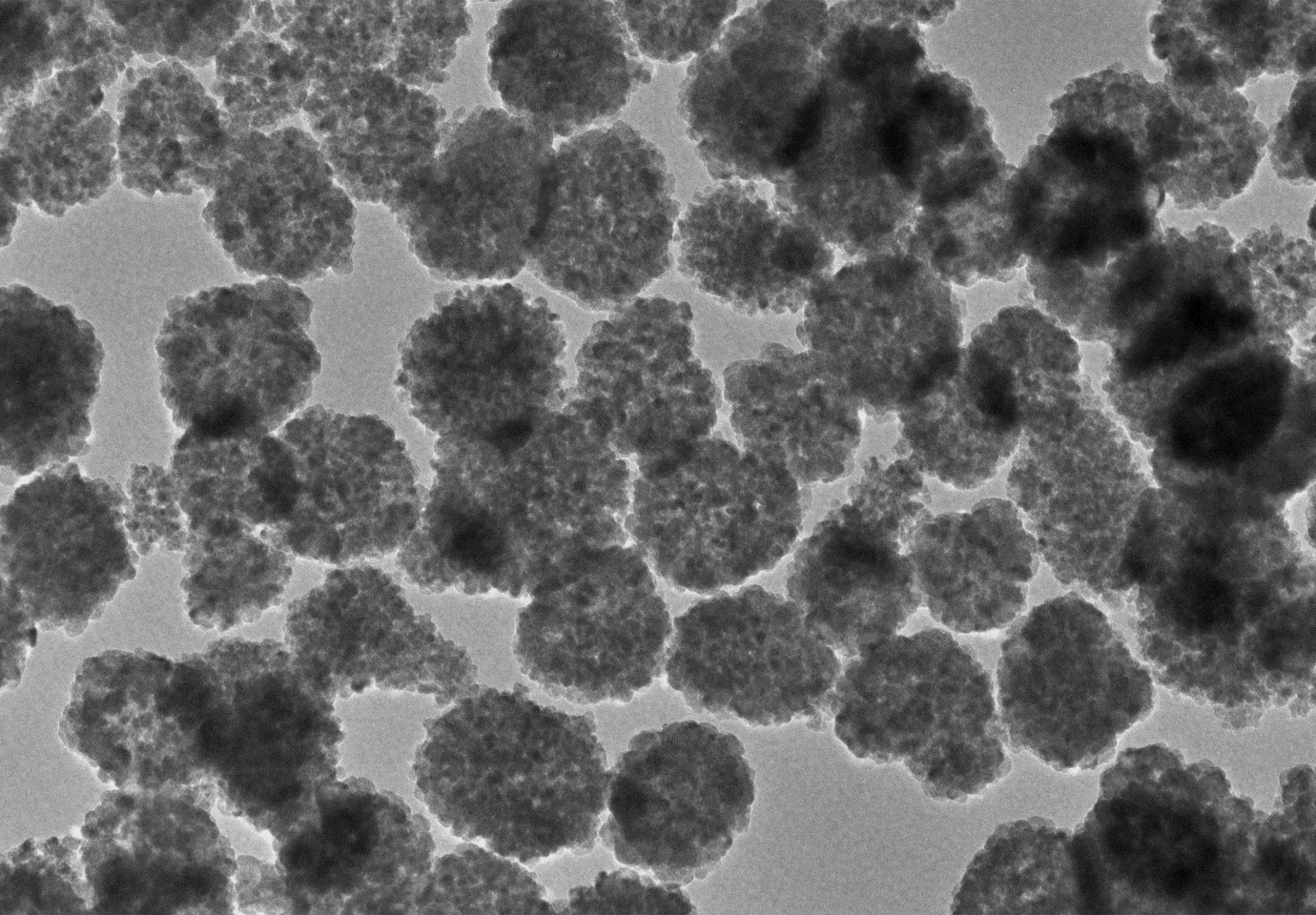


200 nm


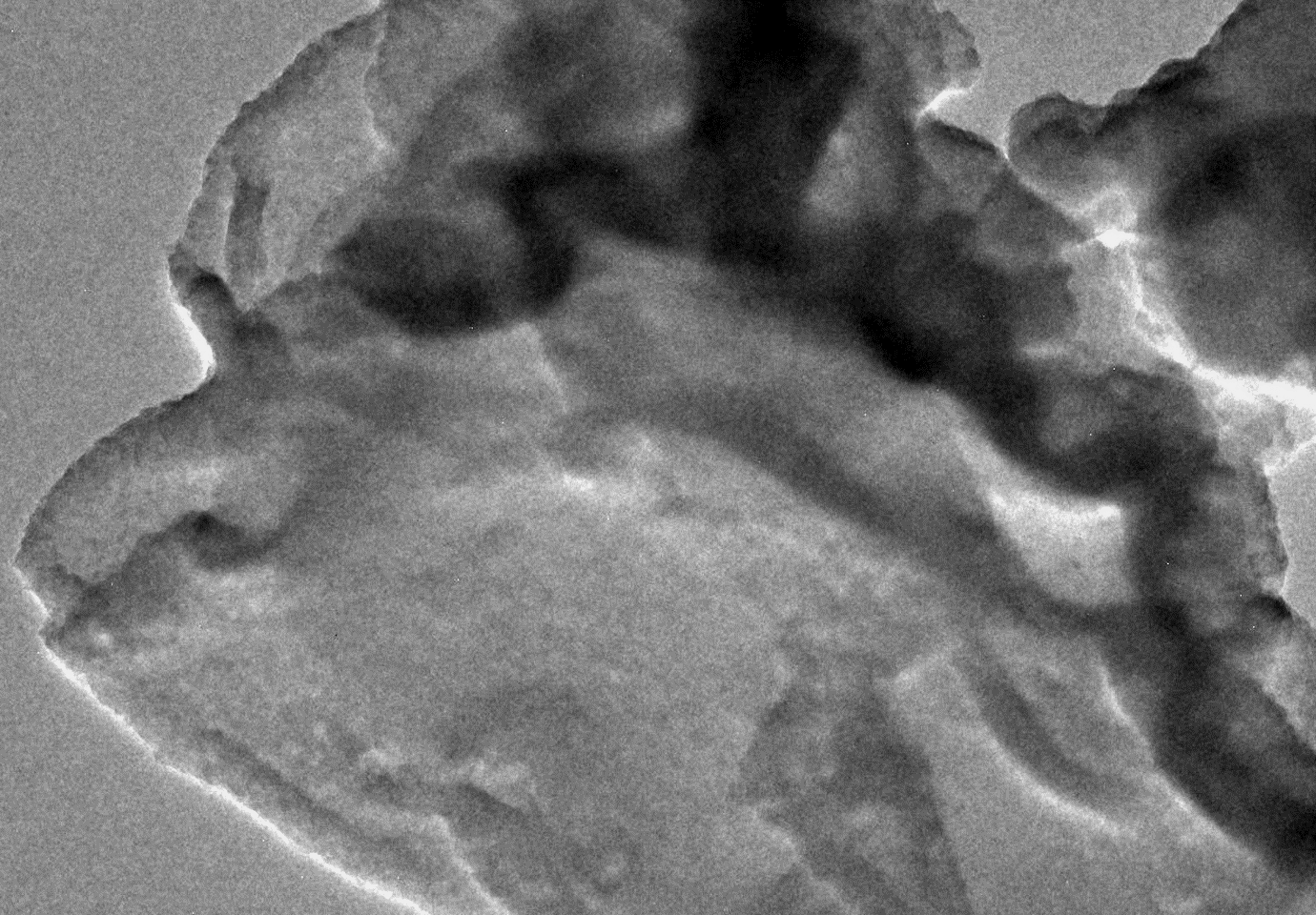


500 nm

e


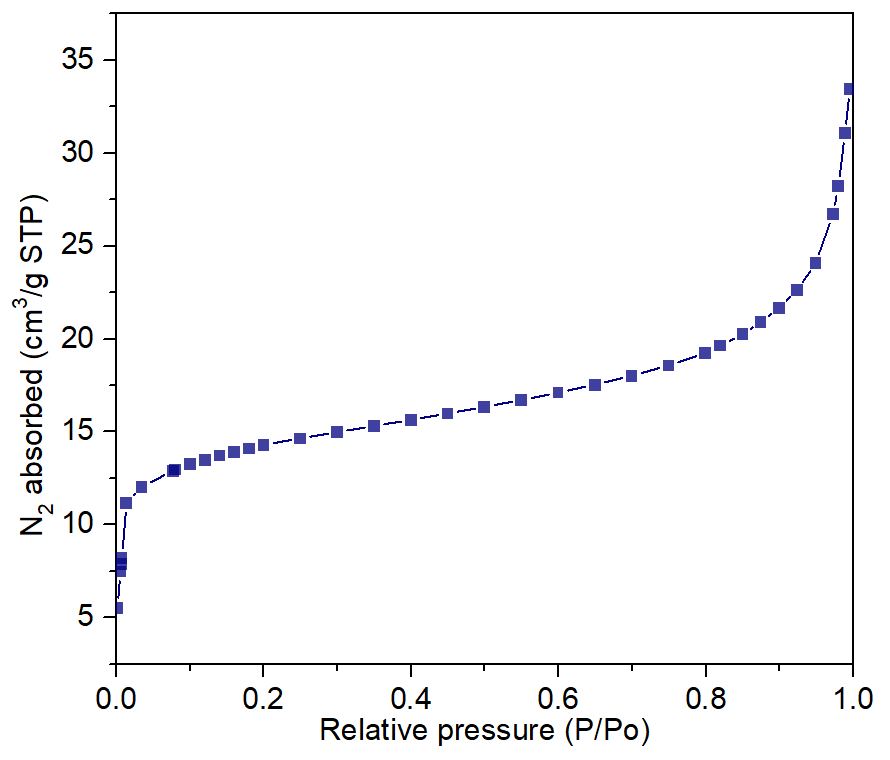

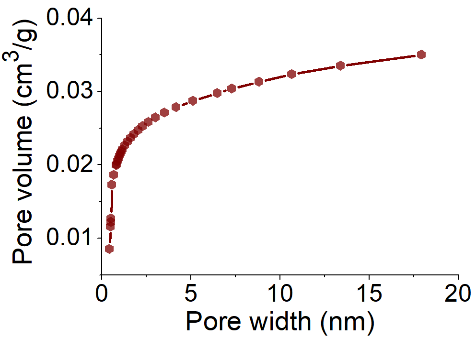


c


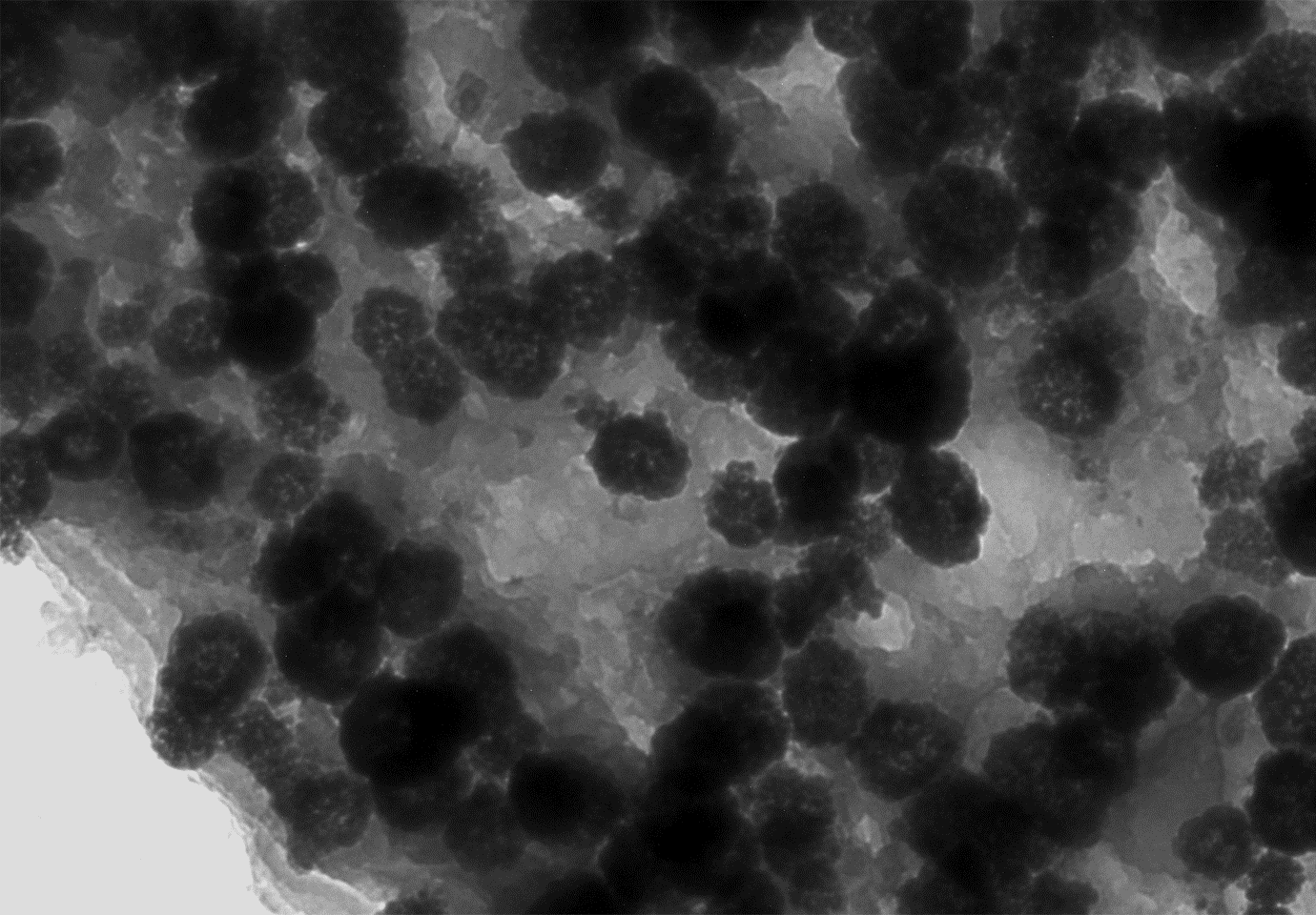


500 nm

S_BET_=66.4569 m²/g

**Supplementary Figure 1 |** **Characterizations of magnetic nanoparticle (MN), porous β-CD polymer (PCDP) and magnetic nanoparticles immobilized porous β-CD polymer (MN-PCDP).** TEM images of **a** MN, **b** PCDP, and **c** MN-PCDP. **d** FT-IR spectra of MN (black), TFT (red), β-CD (blue), PCDP (orange) and MN-PCDP (green). **e** N_2_ adsorption isotherms and cumulative pore volume of MN-PCDP.


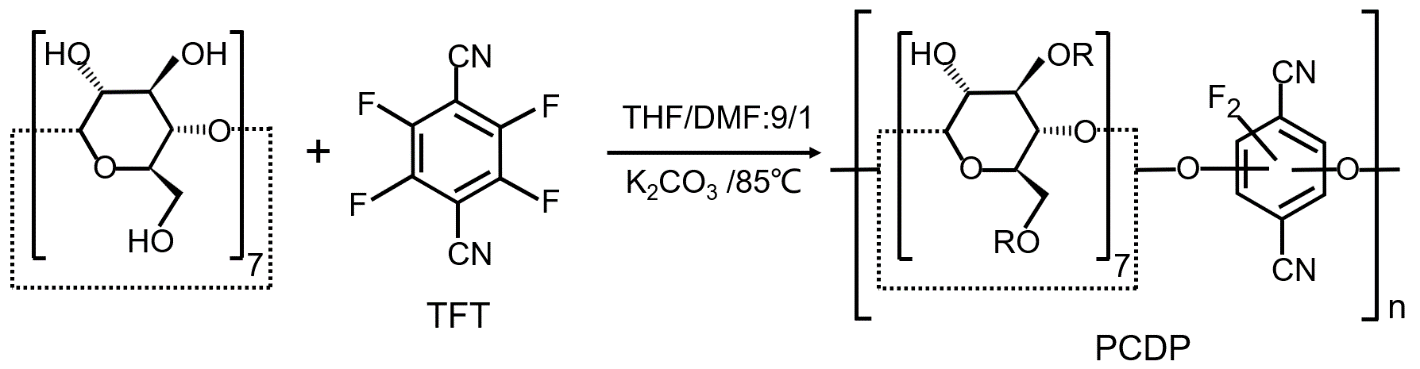


**Supplementary Figure 2 |** schematic about the synthesis of β-CD polymer (PCDP).


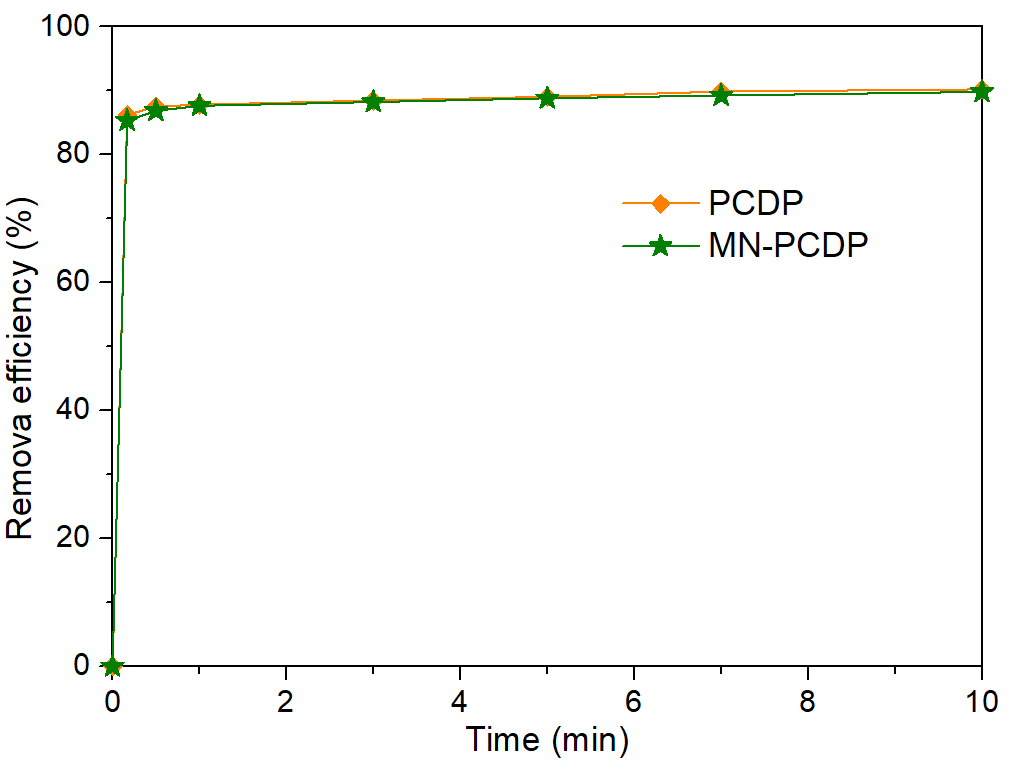

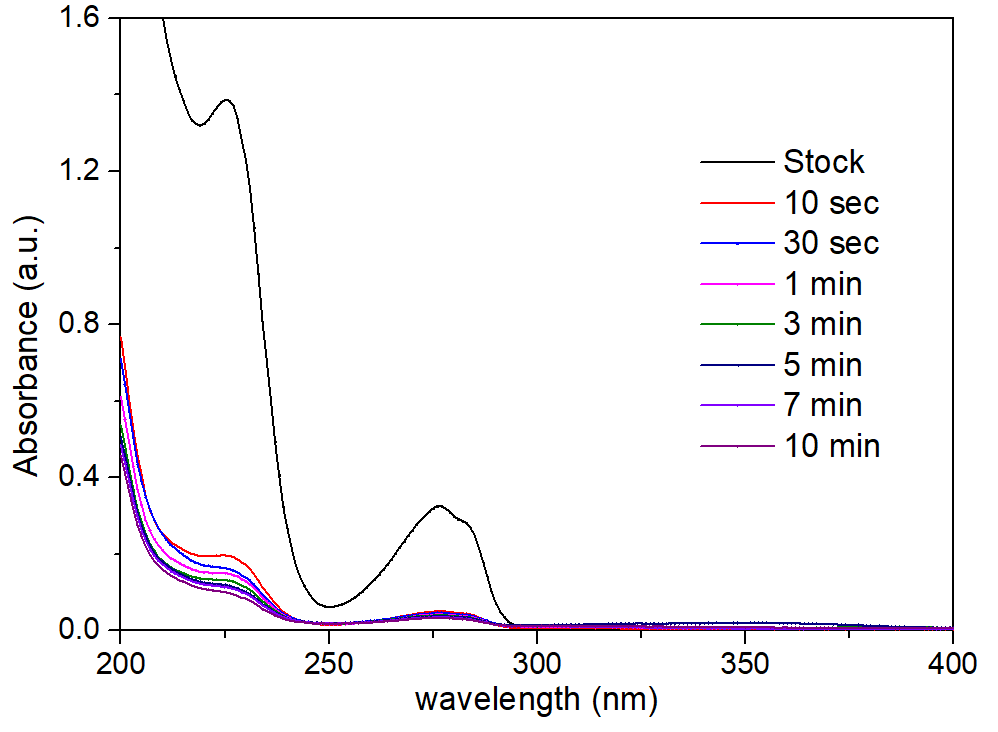

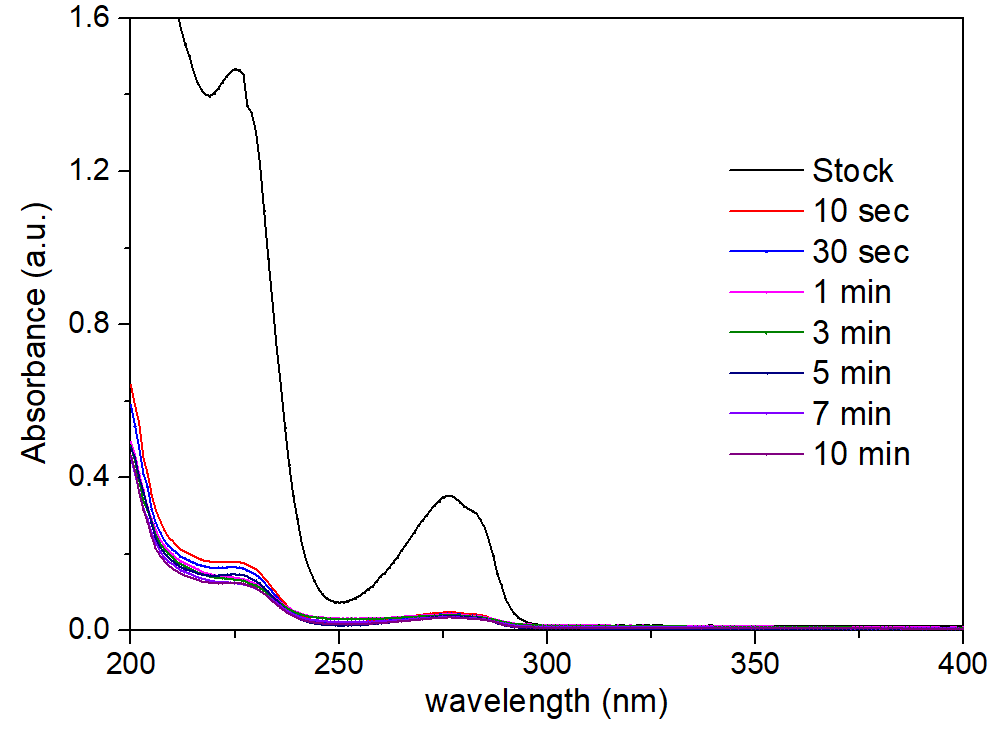


a

b

c

**Supplementary Figure 3 |** Uptake of pollutants by PCDP and MN-PCDP. UV–vis spectra recorded as a function of contact times for BPA (0.1 mM) using **a** PCDP and **b** MN-PCDP as adsorbent with concentration of 1 mg mL^-1^. **c** Time-dependent adsorptions of BPA (0.1 mM) about **a** PCDP and **b** MN-PCDP.


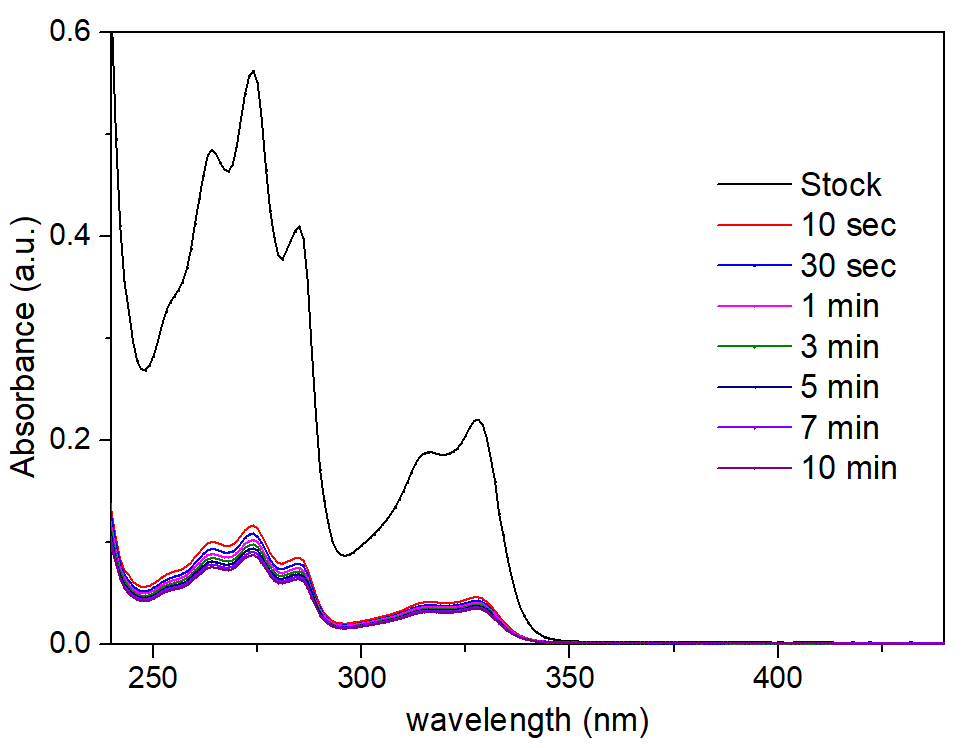

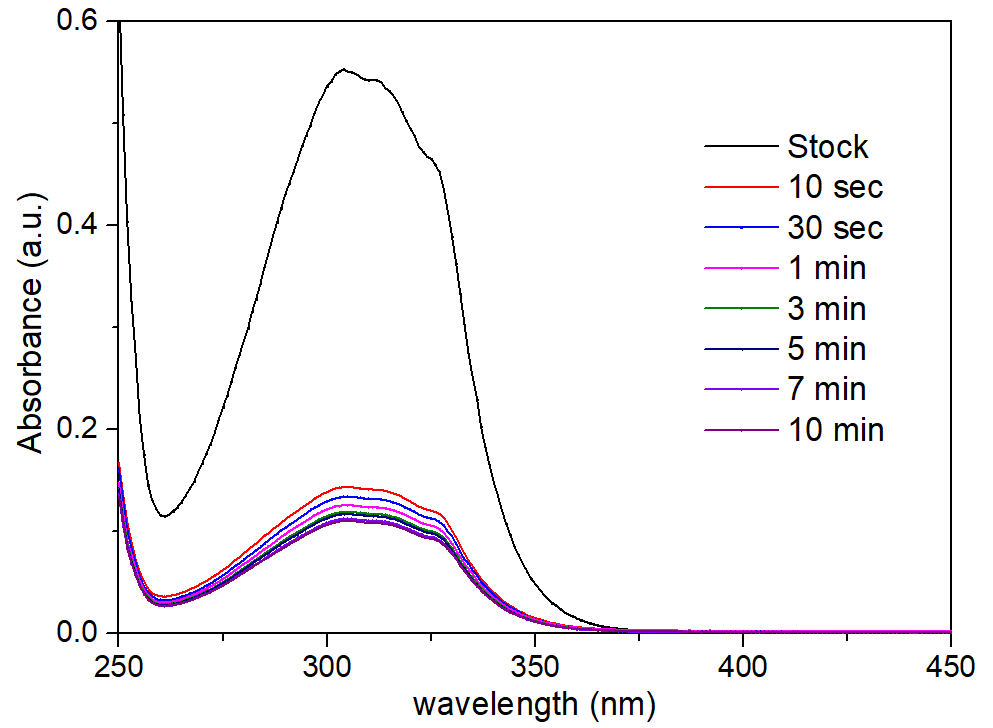

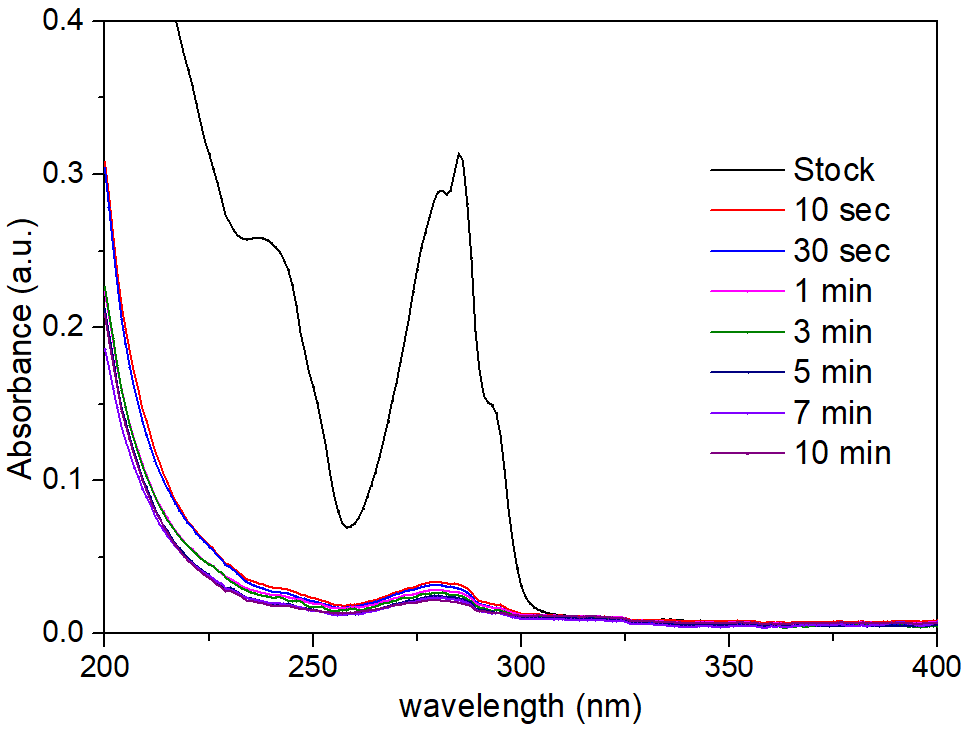

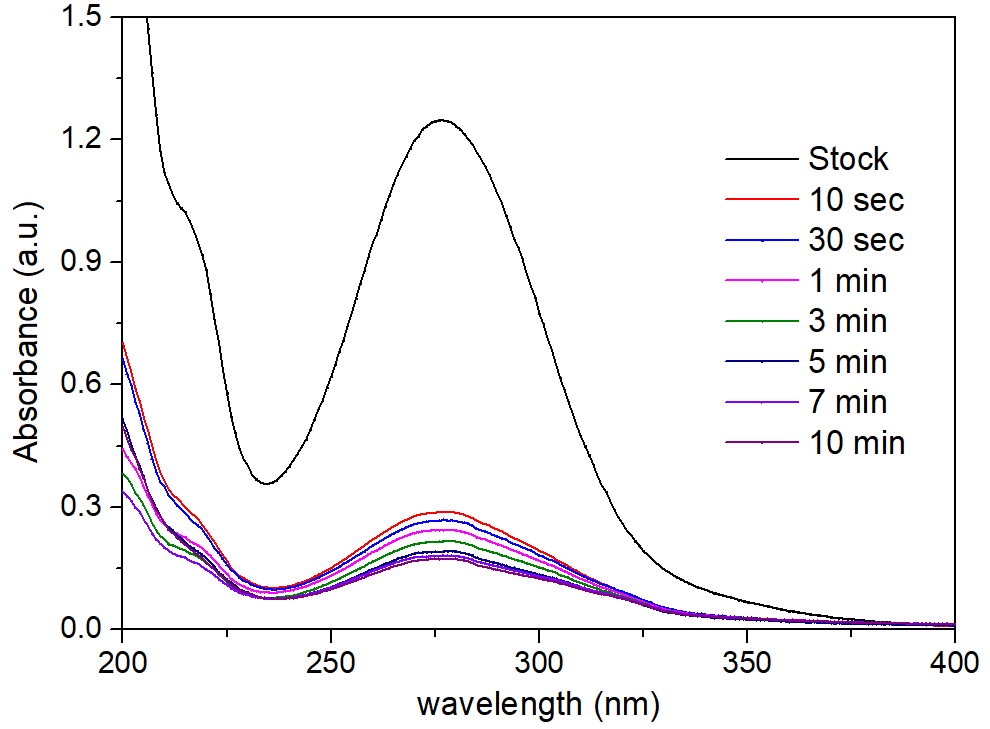

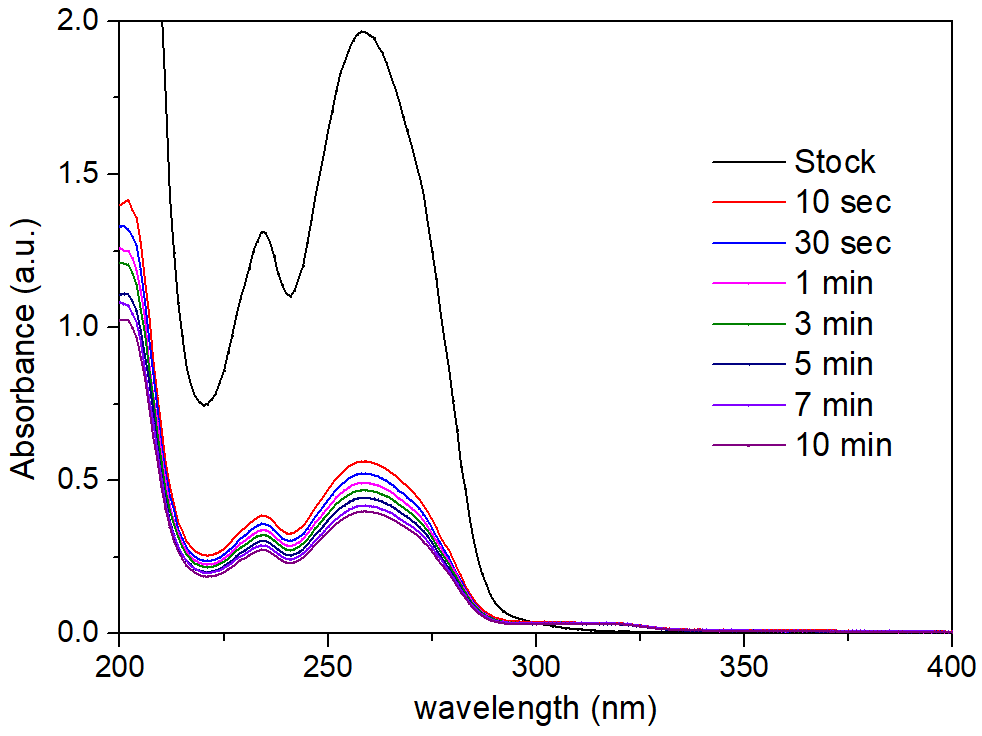

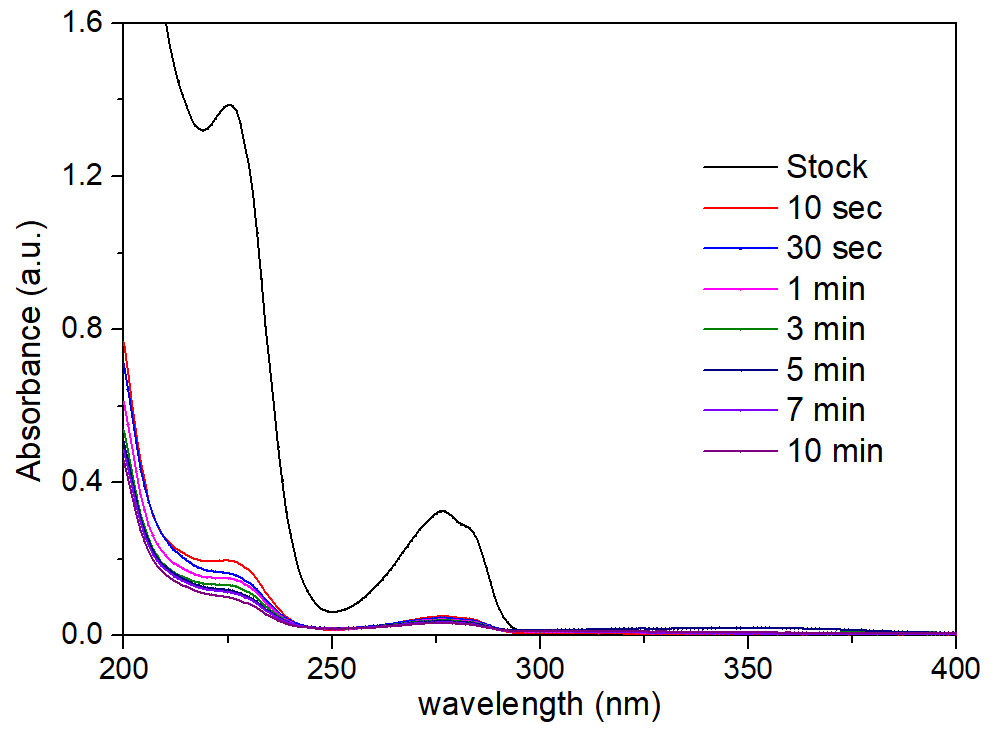


BPS

BPA

Parathion

a

b

c

d

[Carbendazim](javascript:;)

1-NA

2-NO

e

f

**Supplementary Figure 4 |** Uptake of pollutants by MN-PCDP. UV–vis spectra recorded as a function of contact times from **a** BPA, **b** BPS, **c** parathion, **d** carbendazim, **e** 1-NA and **f** 2-NO with 0.1 mM by MN-PCDP (1 mg mL^-1^).

**Supplementary Table 1 |** The removal efficiency of pollutants with contact times using MN-PCDP adsorbent.

| Removal efficiency (%) | BPA | BPS | Parathion | Carbendazim | 1-NA | 2-NO |
| --- | --- | --- | --- | --- | --- | --- |
| 10 s | 85.34 | 72.86 | 79.66 | 89.74 | 74.79 | 80.04 |
| 30 s | 86.86 | 74.95 | 80.94 | 90.59 | 76.52 | 81.50 |
| 1 min | 87.65 | 76.47 | 82.44 | 91.37 | 77.99 | 82.54 |
| 3 min | 88.24 | 77.74 | 84.23 | 92.07 | 79.19 | 83.33 |
| 5 min | 88.82 | 79.04 | 86.15 | 92.68 | 79.61 | 84.06 |
| 7 min | 89.27 | 80.39 | 86.35 | 92.99 | 80.47 | 84.66 |
| 10 min | 89.92 | 81.33 | 86.96 | 93.58 | 80.82 | 85.20 |


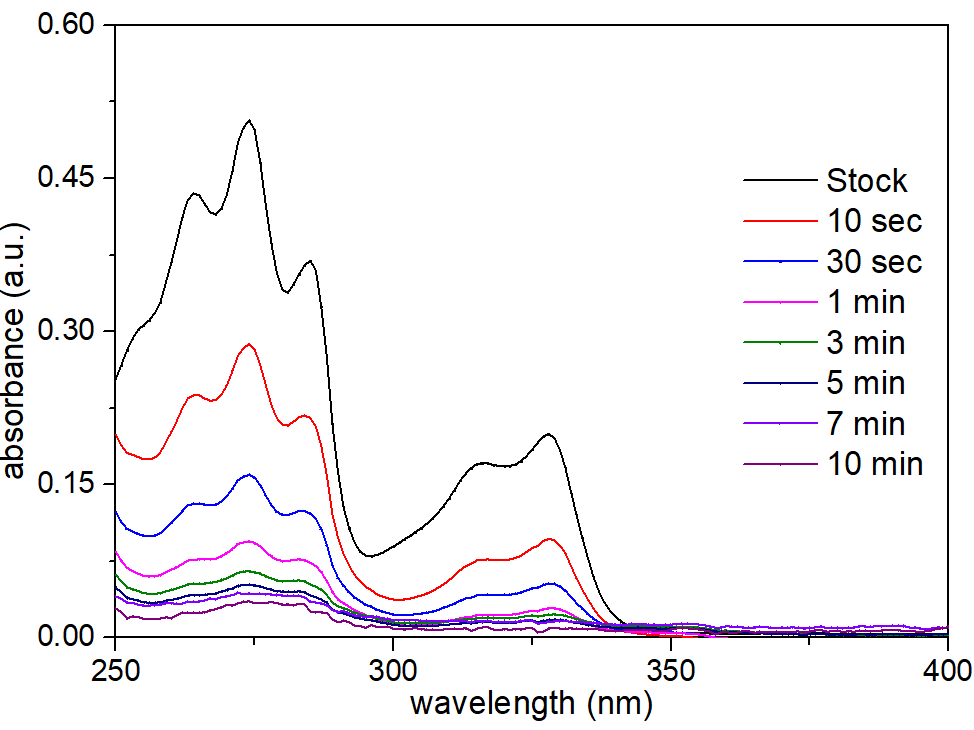

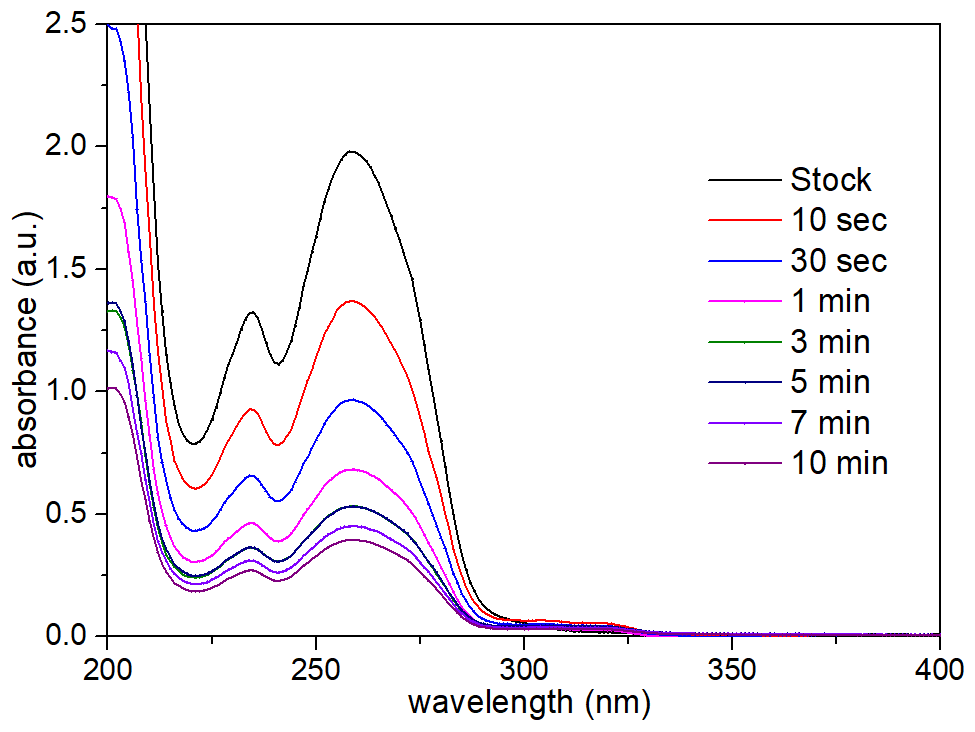

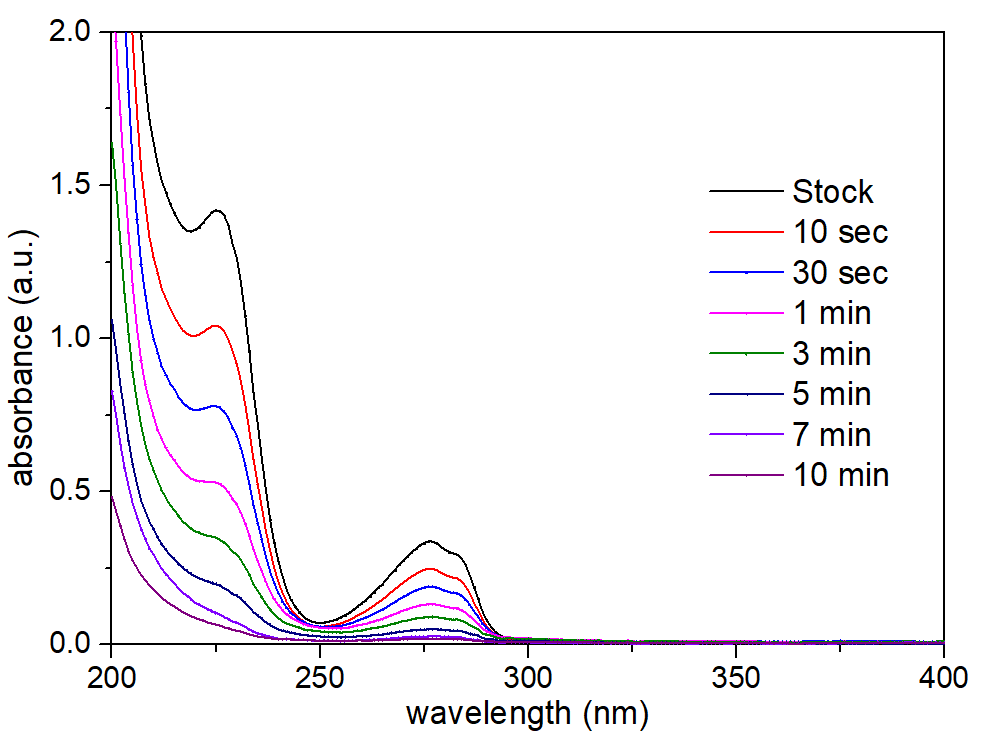

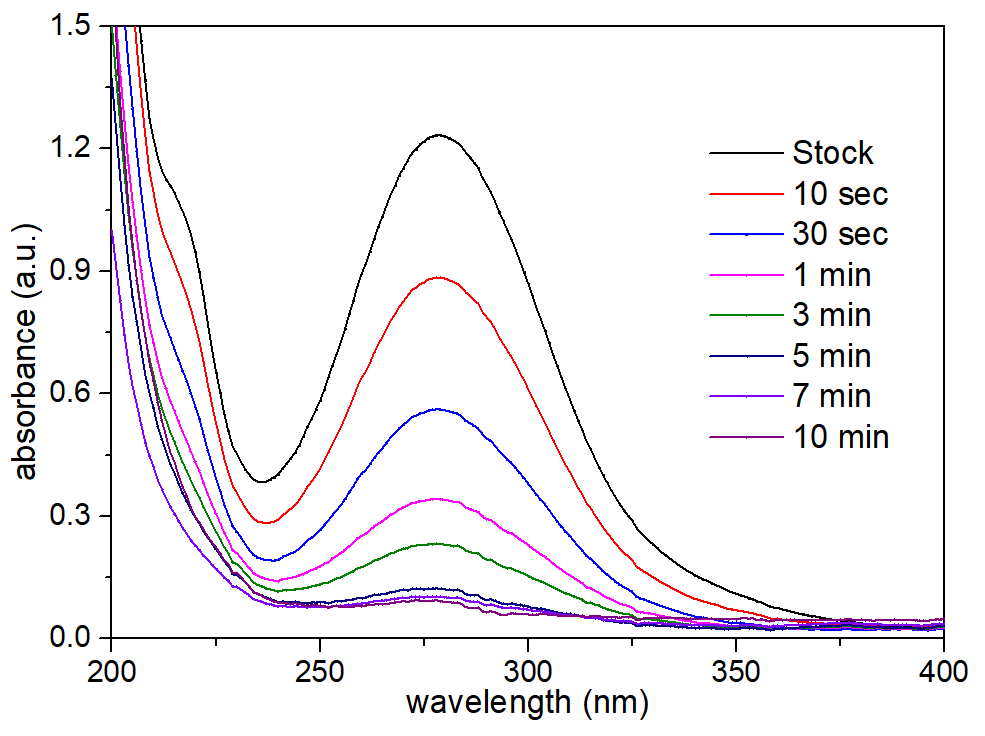

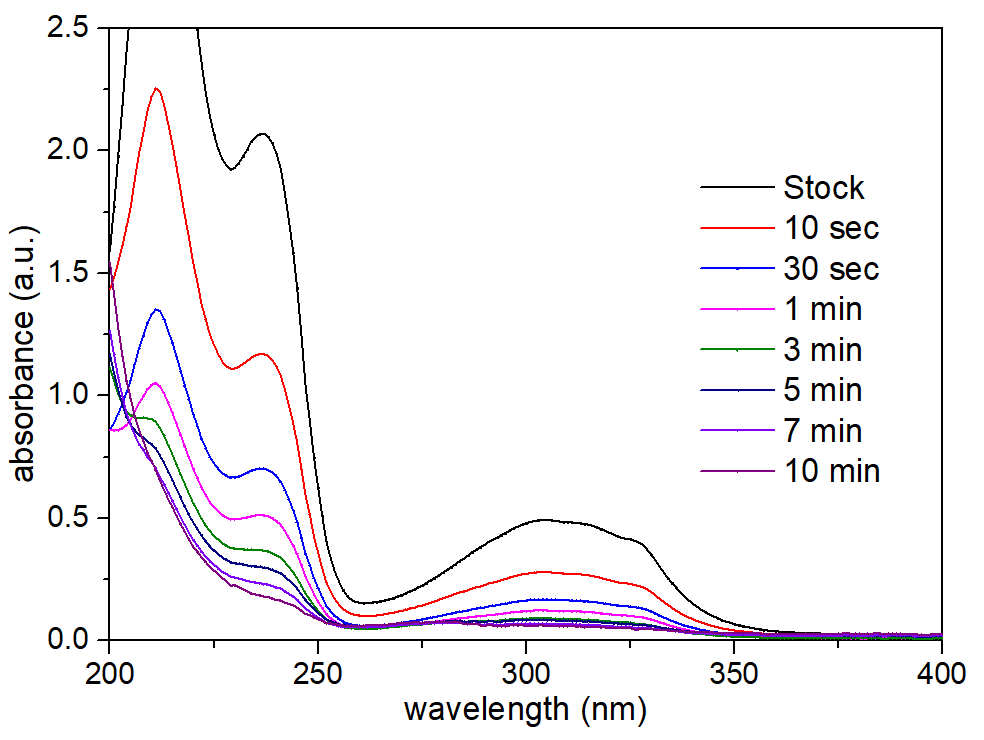

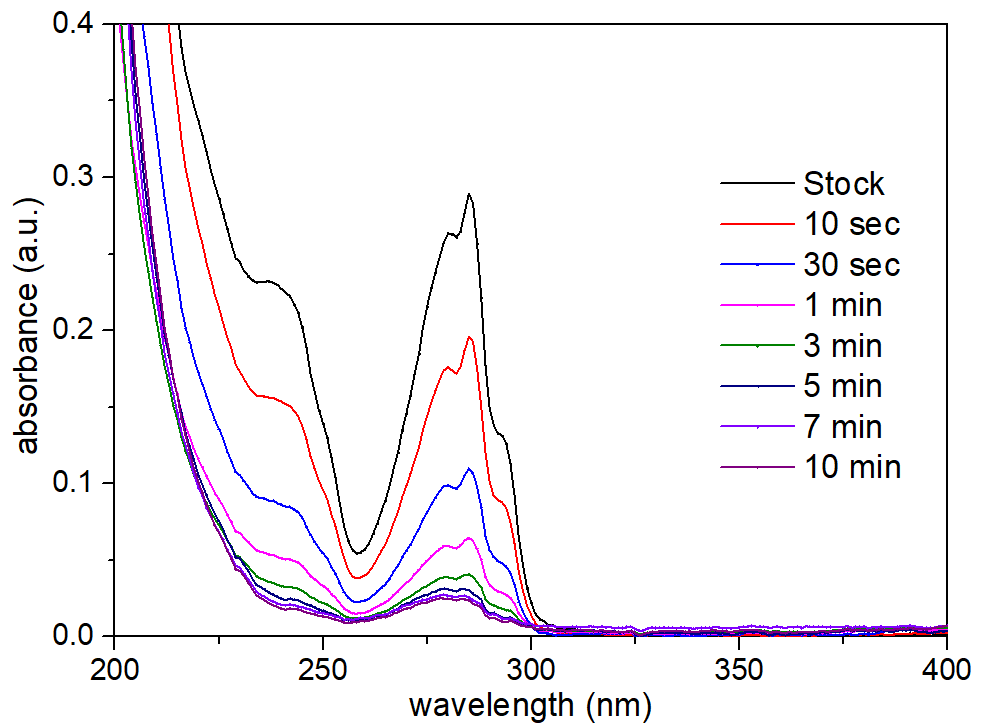


BPA-NAC

a

BPS-NAC

b

1-NA-NAC

2-NO-NAC

e

Parathion-NAC

[Carbendazim](javascript:;)-NAC

c

d

f

**Supplementary Figure 5 |** Uptake of pollutants by NAC. UV–vis spectra recorded as a function of contact times from **a** BPA, **b** BPS, **c** parathion, **d** carbendazim, **e** 1-NA and **f** 2-NO with 0.1 mM by NAC (1 mg mL^-1^) in 20 mL pollutants.


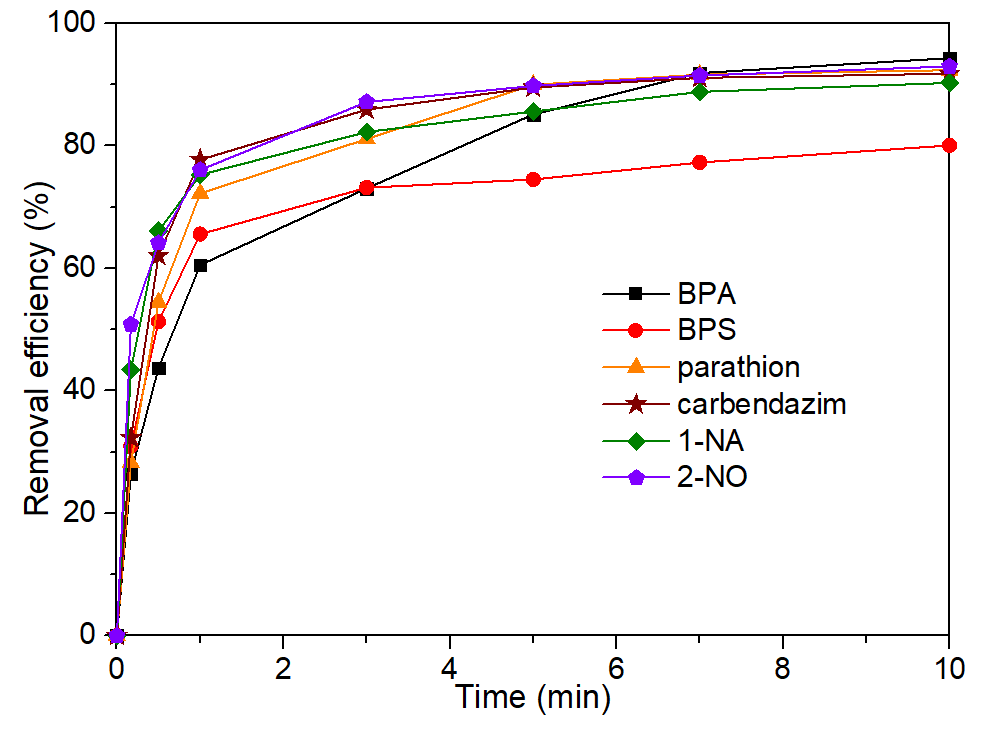


**Supplementary Figure 6 |** Uptake of pollutants by NAC. Time-dependent adsorption of **a** BPA, **b** BPS, **c** parathion, **d** carbendazim, **e** 1-NA and **f** 2-NO with 0.1 mM by NAC (1 mg mL^-1^).

**Supplementary Table 2 |** The removal efficiency of pollutants with contact times using NAC.

| Removal efficiency (%) | BPA | BPS | Parathion | Carbendazim | 1-NA | 2-NO |
| --- | --- | --- | --- | --- | --- | --- |
| 10 s | 26.45 | 30.93 | 28.26 | 32.30 | 43.45 | 50.87 |
| 30 s | 43.71 | 51.29 | 54.44 | 62.05 | 66.07 | 64.10 |
| 1 min | 60.54 | 65.57 | 72.22 | 77.71 | 75.28 | 76.06 |
| 3 min | 73.05 | 73.17 | 81.11 | 85.92 | 82.27 | 87.14 |
| 5 min | 85.13 | 73.21 | 90.00 | 89.52 | 85.60 | 89.78 |
| 7 min | 91.88 | 77.27 | 91.63 | 91.10 | 88.82 | 91.47 |
| 10 min | 94.31 | 80.07 | 92.35 | 91.77 | 90.28 | 92.98 |


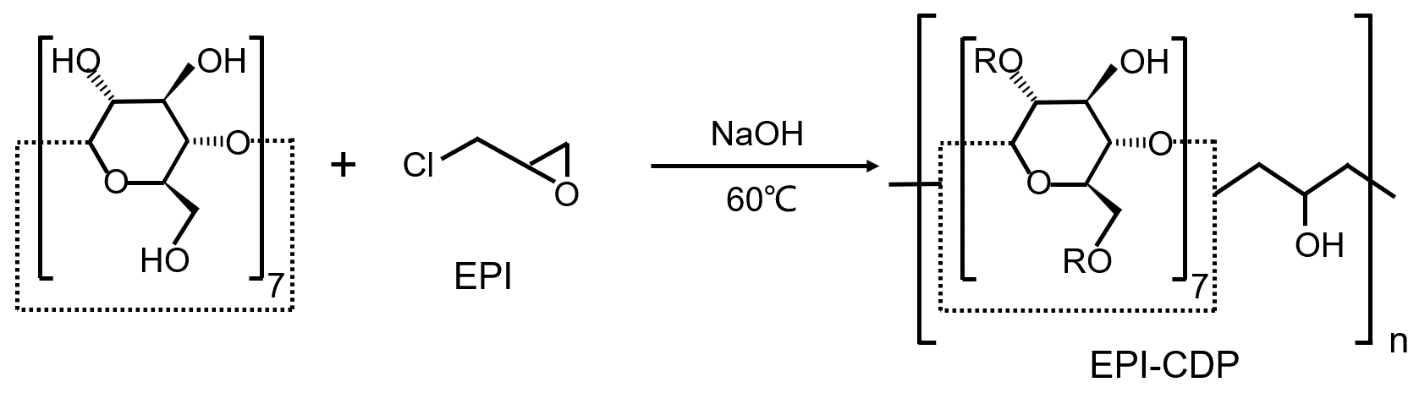


**Supplementary Figure 7 |** schematic about the synthesis of β-CD polymer crosslinked by EPI (EPI-CDP).


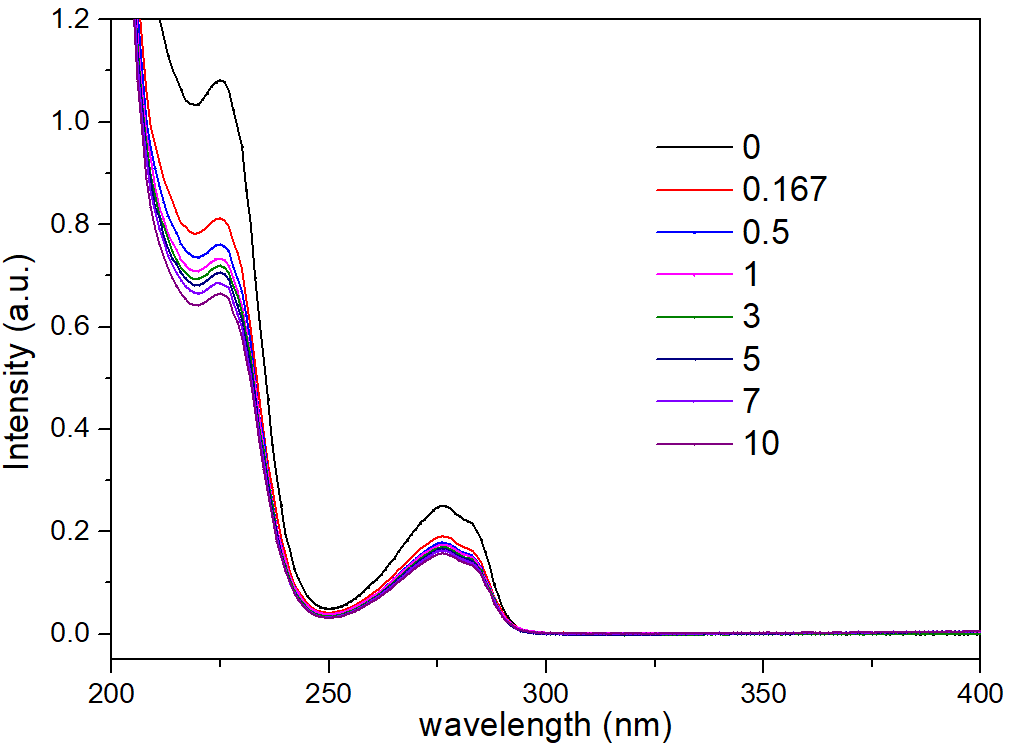

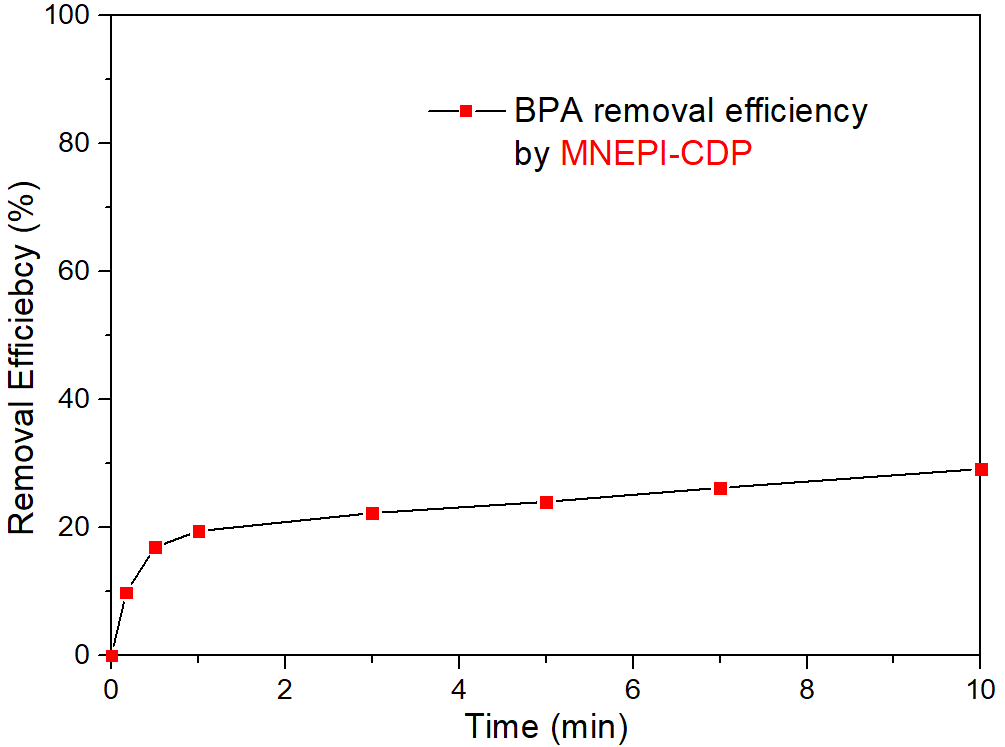


a

b

**Supplementary Figure 8** | Uptake of pollutant by MNEPI-CDP. UV–vis spectra and removal efficiency recorded at different contact times of bisphenol A solution (0.1 mM) by MNEPI-CDP (1 mg mL^-1^).


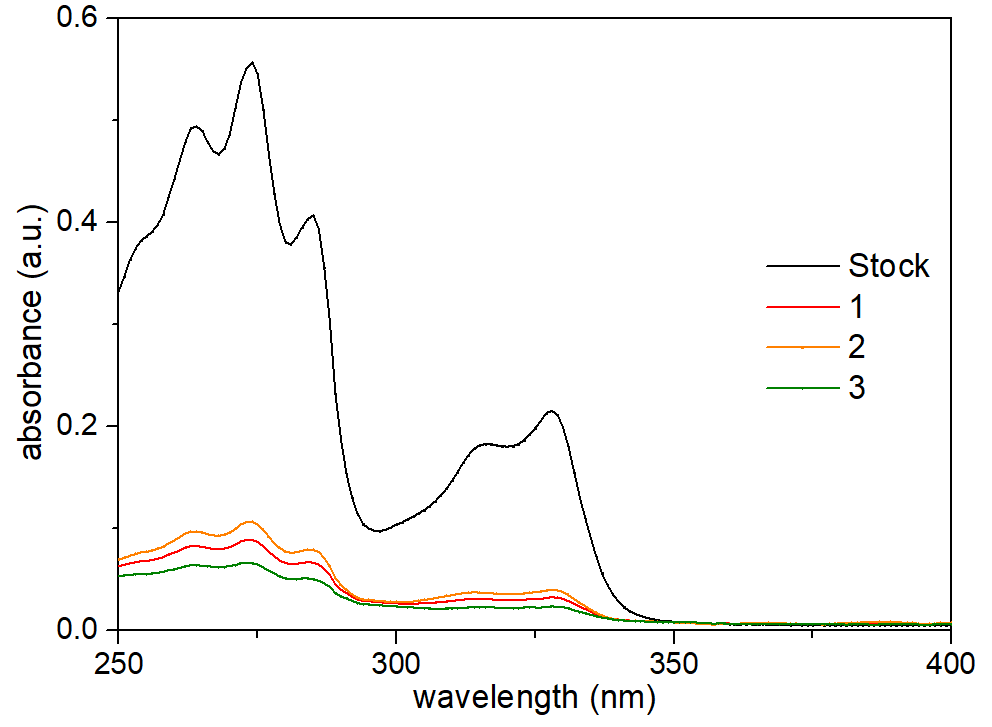

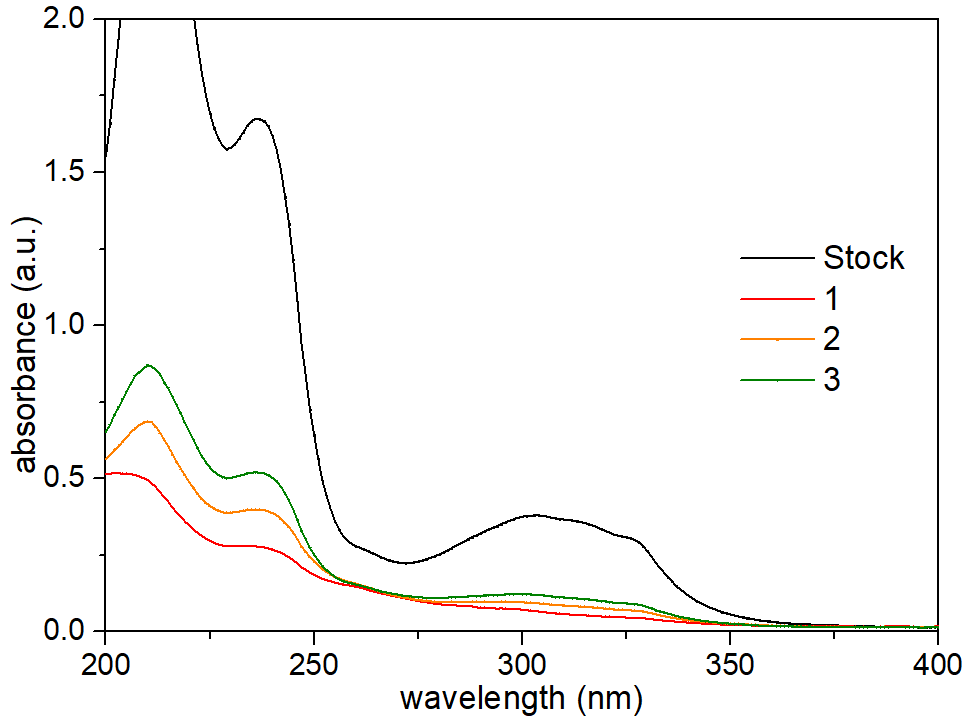

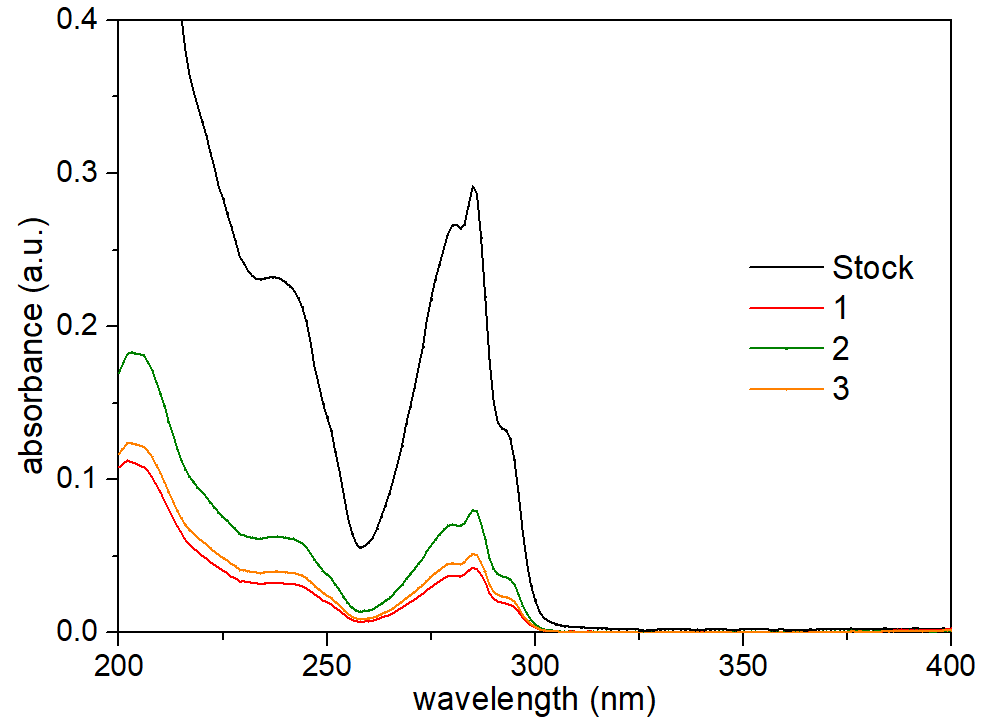

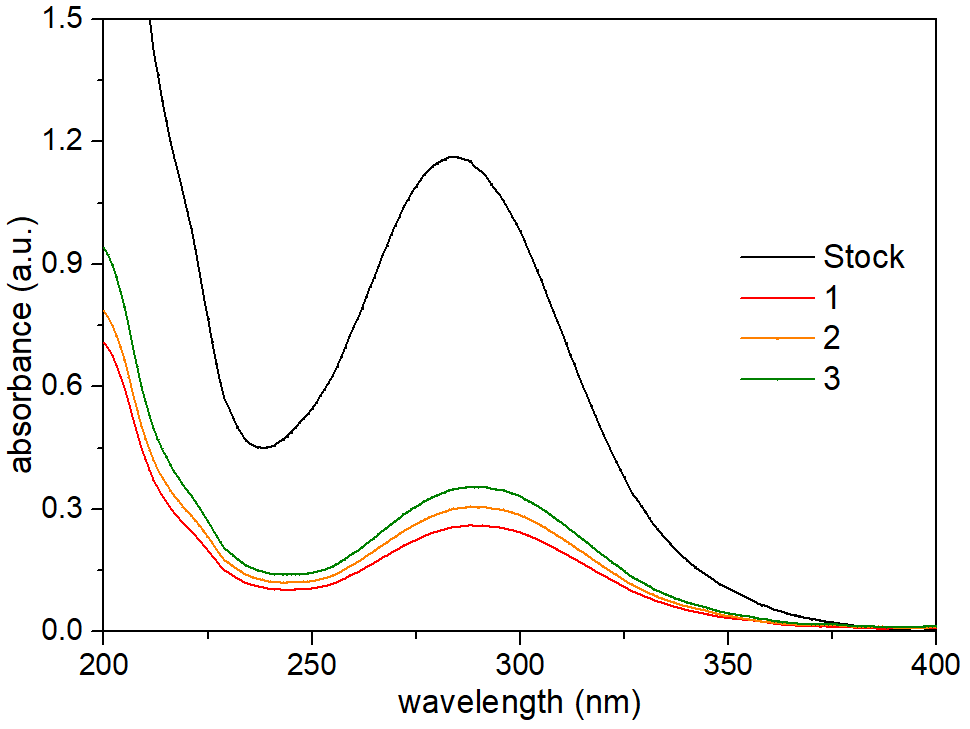

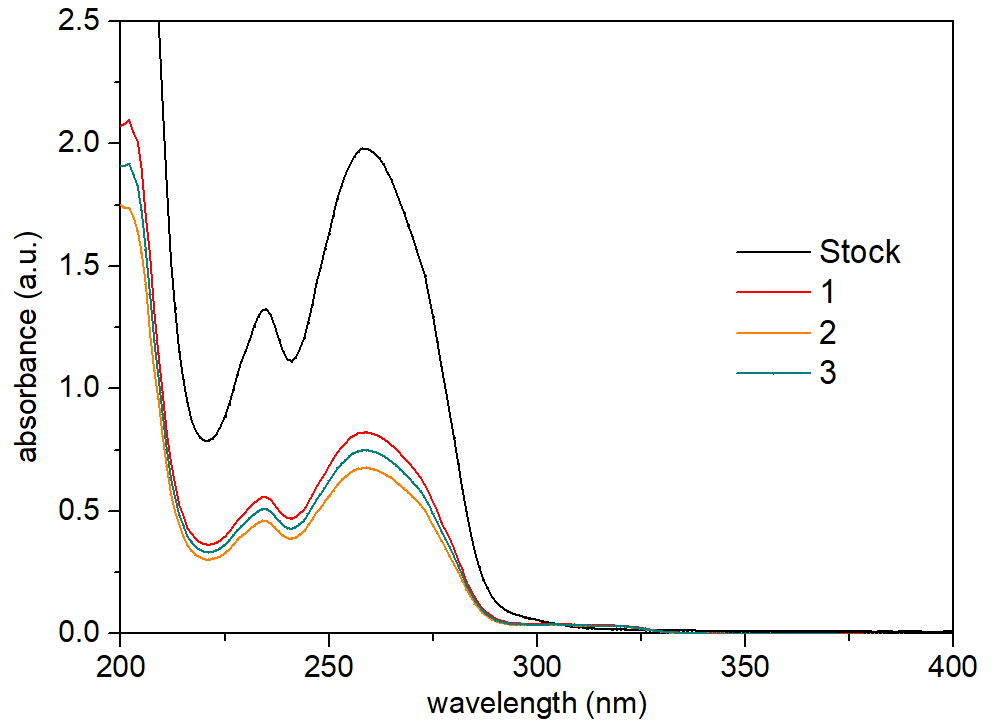

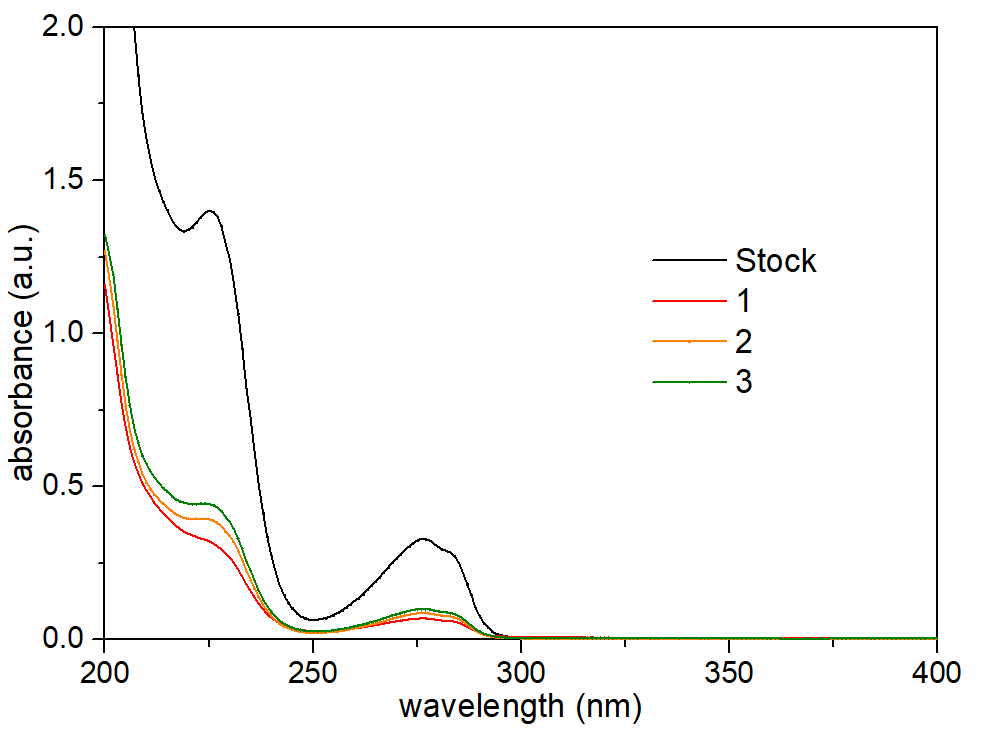


Parathion

BPS

BPA

1-NA

2-NO

[Carbendazim](javascript:;)

a

b

c

d

e

f

**Supplementary Figure 9 |** Uptake of pollutants by a thin layer of MN-PCDP. UV–vis spectra recorded as a function of triplicate absorption experiments from 5 mL of **a** BPA, **b** BPS, **c** parathion, **d** carbendazim, **e** 1-NA and **f** 2-NO with 0.1 mM through a thin layer of MN-PCDP (5 mg) in 30 s.


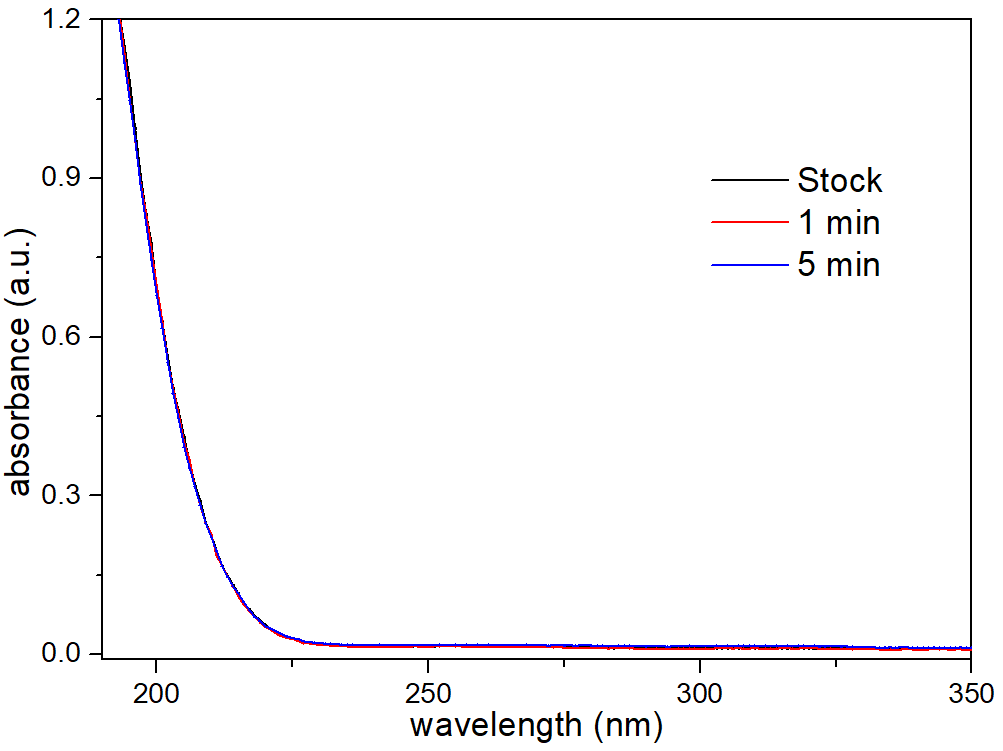

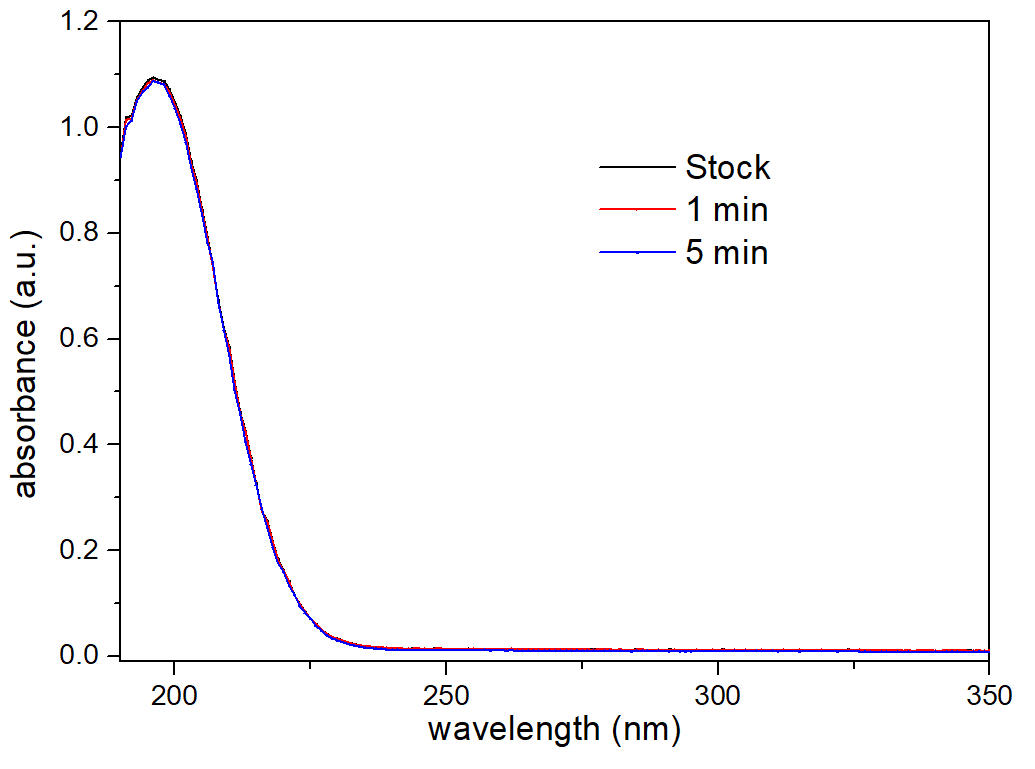


a

DMF


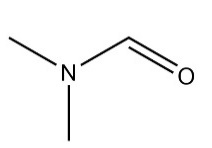

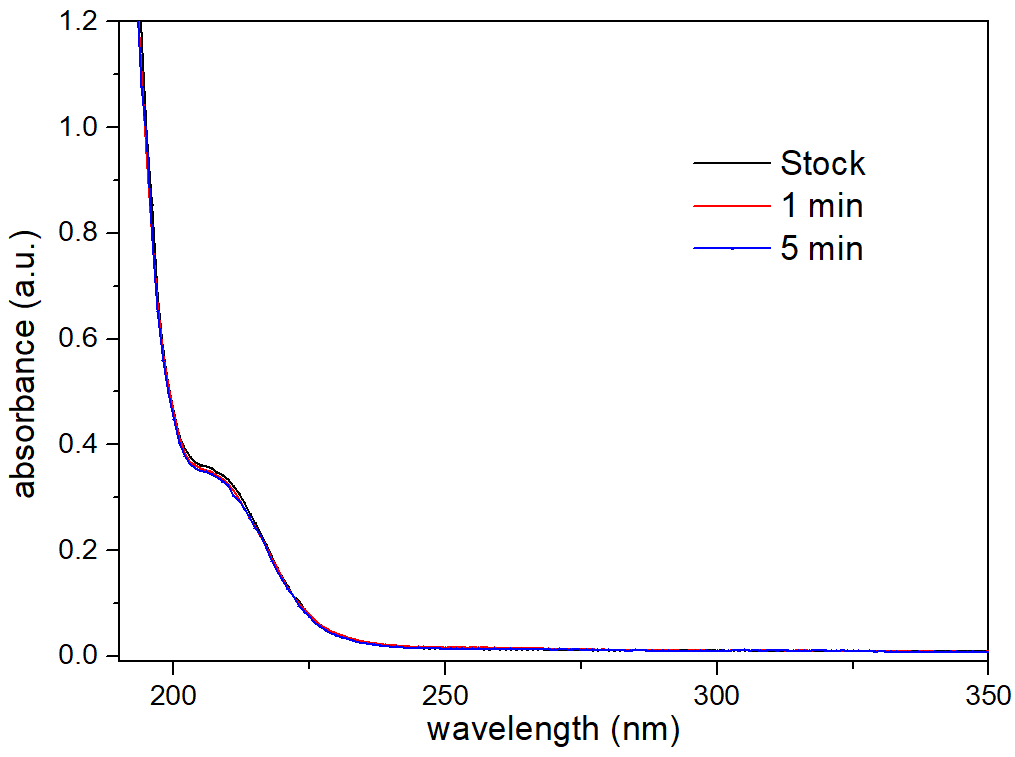


PFOA

b


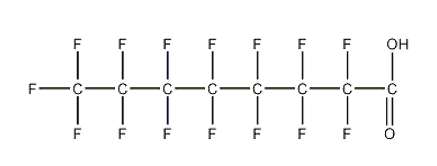

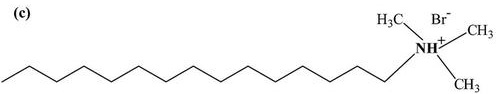

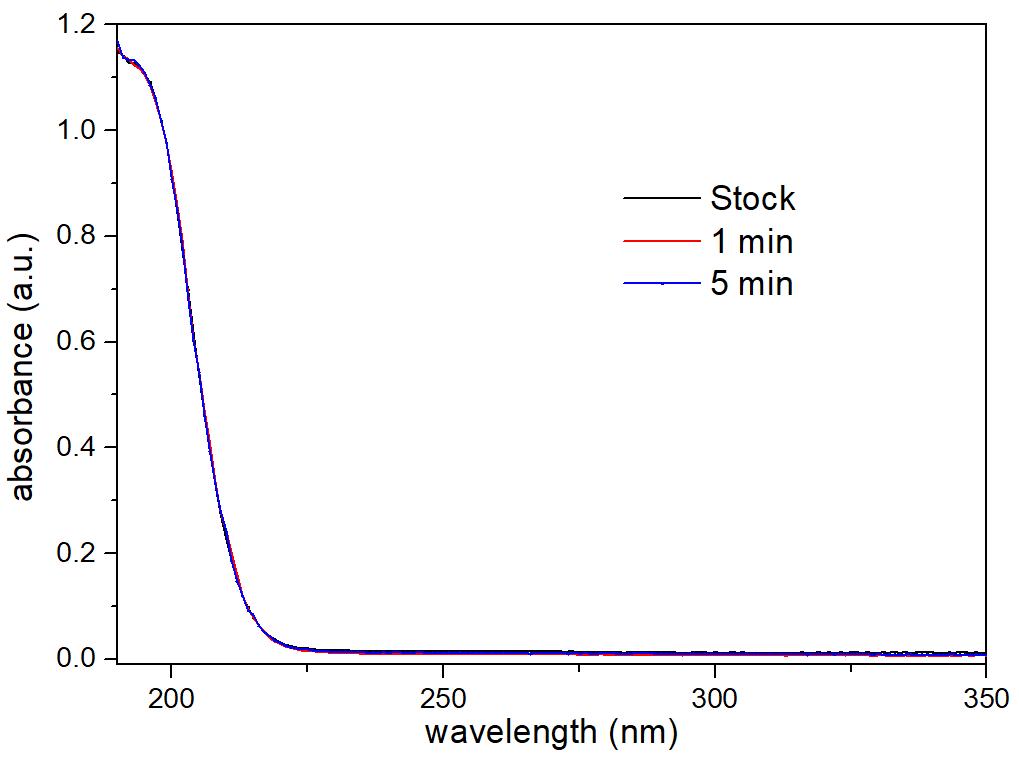


c

CTAB


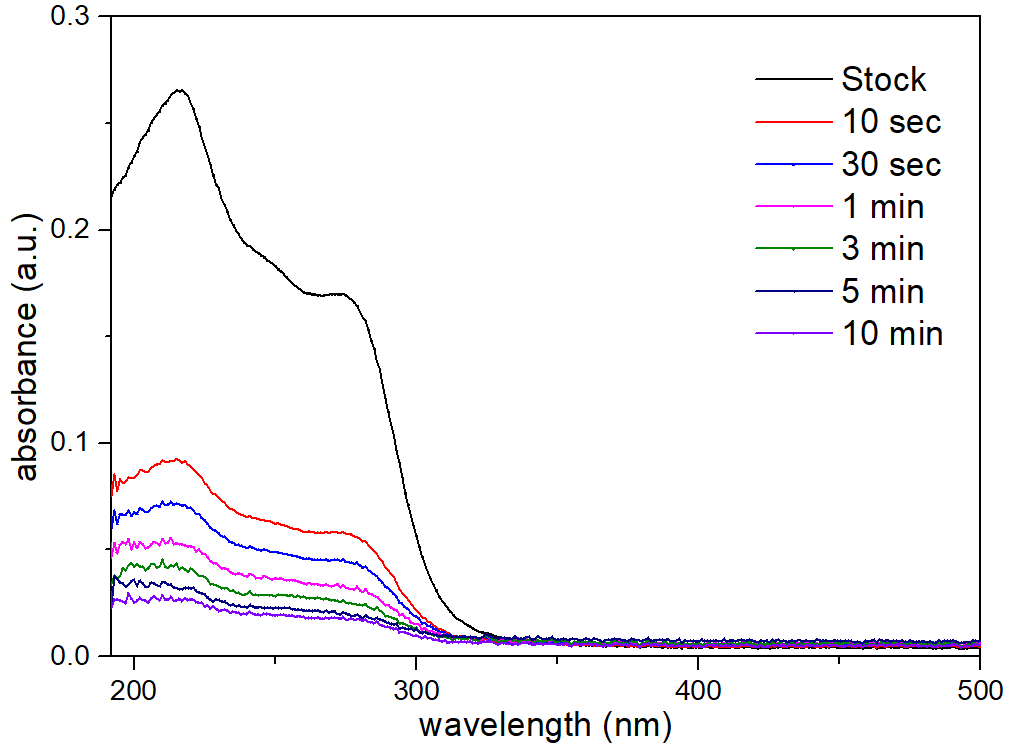


e

TMTD


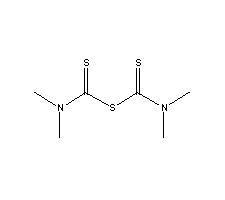

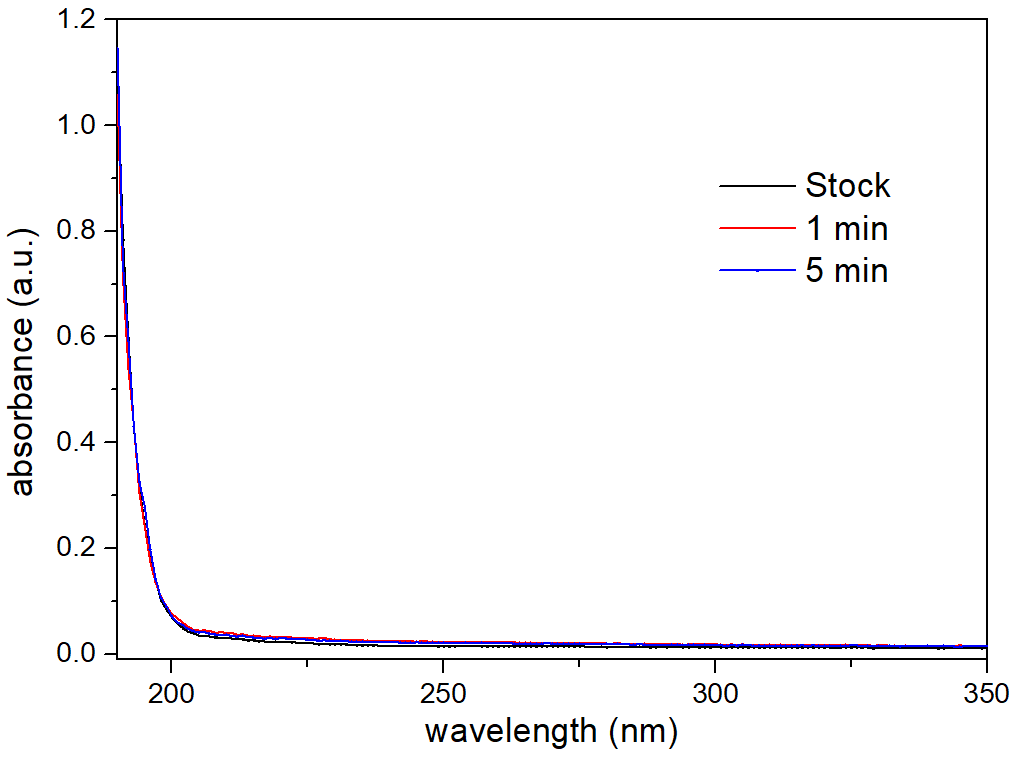


d

CCC


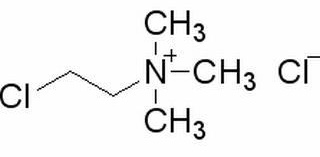


HMTA


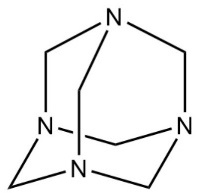


f

**Supplementary Figure 10** | Uptake of pollutant by MN-PCDP. UV–vis spectra at different contact times of DMF (0.1 mM, **a**), PFOA (1 mM, **b**), CTAB (0.1 mM, **c**), CCC (1 mM, **d**), TMTD (0.1 mM, **e**) and HMTA (1 mM, **f**) with the adsorbent (1 mg mL^-1^).


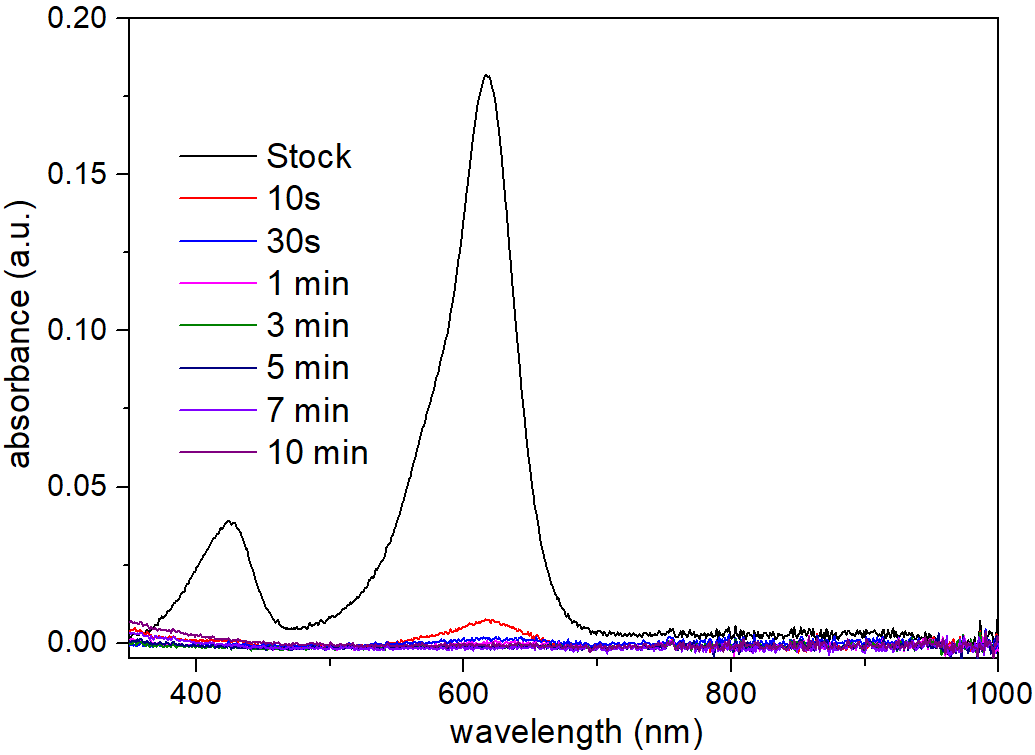


a

MG


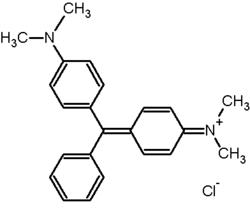

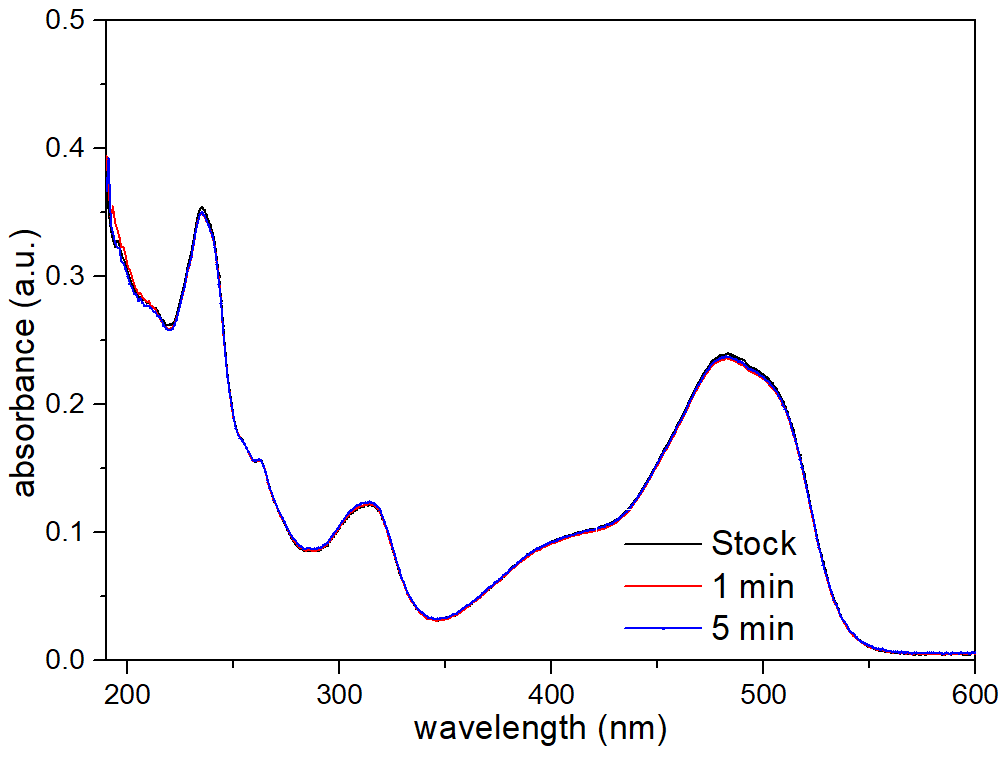


b


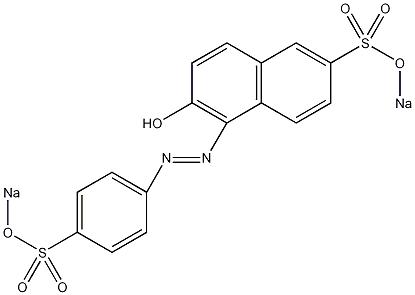


SY

**Supplementary Figure 11** | Uptake of pollutant by MN-PCDP. UV–vis spectra and removal efficiency recorded at different contact times of MG (0.01 mM, **a**) and SY (0.01 mM, **b**) with the adsorbent (1 mg mL^-1^).


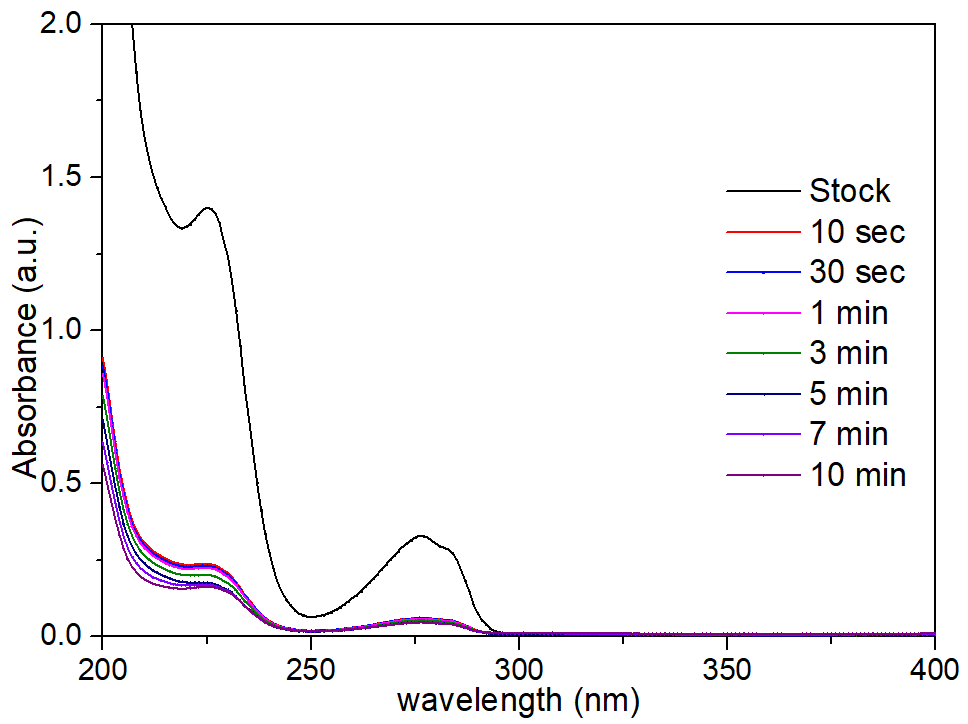

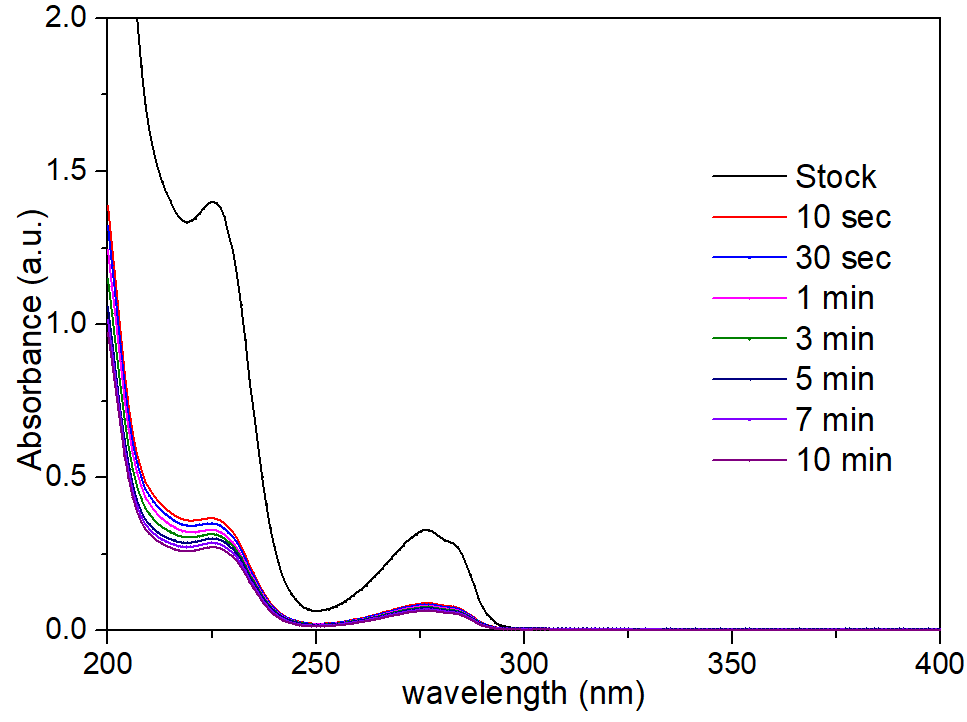

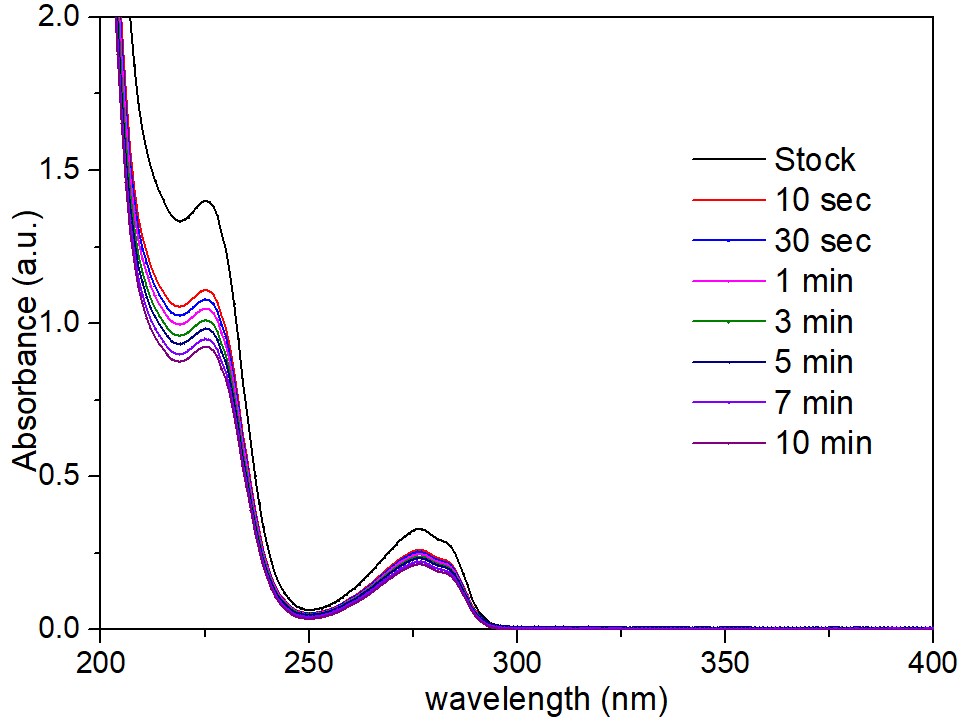

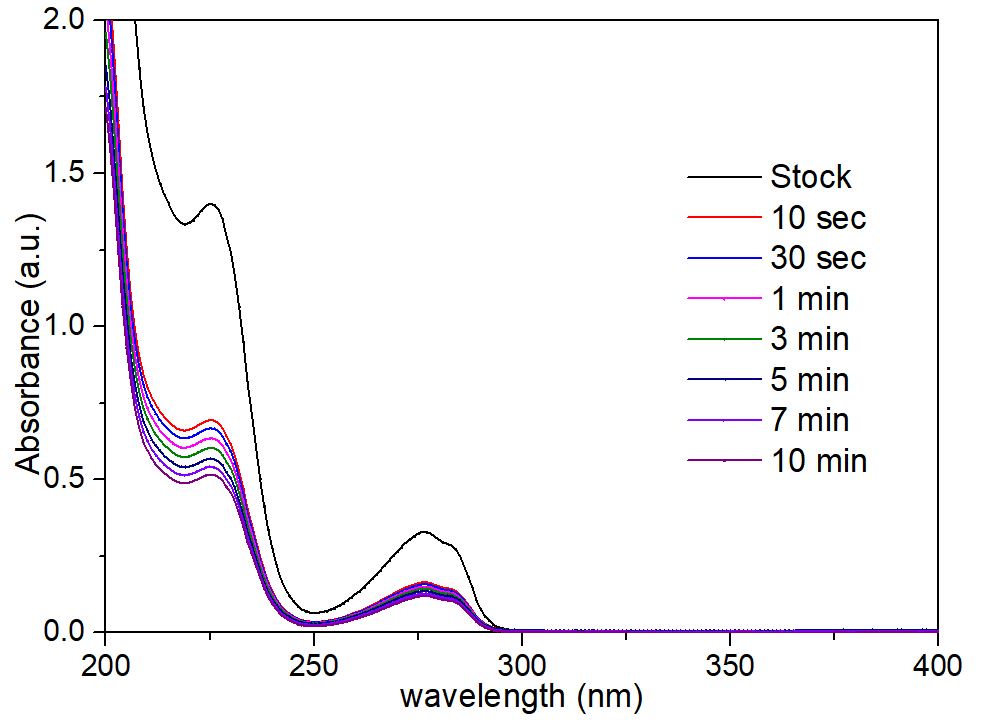


0.1 mg mL^-1^

0.25 mg mL^-1^

0.5 mg mL^-1^

0.75 mg mL^-1^

a

b

c

d

**Supplementary Figure 12 |** Uptake of pollutants by MN-PCDP. UV–vis spectra recorded as a function of contact times of BPA (0.1 mM, 100 mL) using different dosage of MN-PCDP.

**Supplementary Table 3 |** The removal efficiency of BPA with contact times using different dosage MN-PCDP

| Removal efficiency (%) | 0.1 mg/mL | 0.25 mg/mL | 0.5 mg/mL | 0.75 mg/mL | 1 mg/mL |
| --- | --- | --- | --- | --- | --- |
| 10 s | 20.78 | 50.05 | 73.65 | 81.07 | 84.85 |
| 30 s | 22.97 | 51.96 | 76.95 | 81.58 | 86.26 |
| 1 min | 25.12 | 54.29 | 78.50 | 82.12 | 87.09 |
| 3 min | 27.47 | 57.25 | 78.98 | 83.76 | 87.94 |
| 5 min | 29.09 | 60.37 | 79.36 | 85.57 | 88.43 |
| 7 min | 32.42 | 63.40 | 79.74 | 85.70 | 89.17 |
| 10 min | 35.07 | 65.65 | 80.01 | 85.83 | 89.82 |


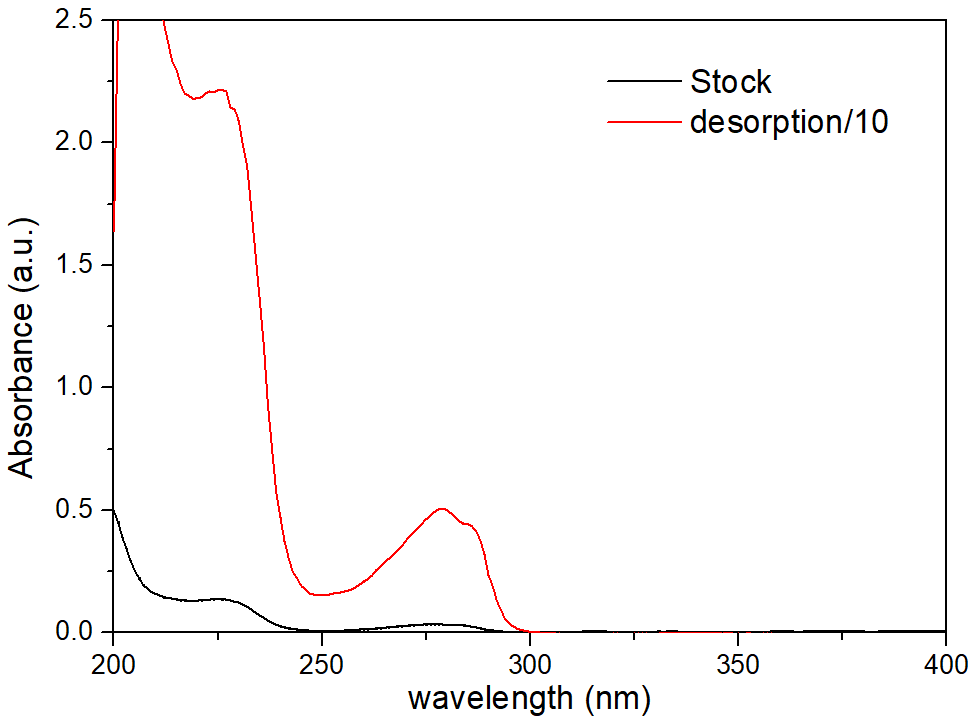


**Supplementary Figure 13 |** Desorption of BPA (0.01 mM, 100 mL) by MN-PCDP. UV–vis spectra of BPA in initial (black) and after enrichment (red) using MN-PCDP (1 mg mL^-1^).


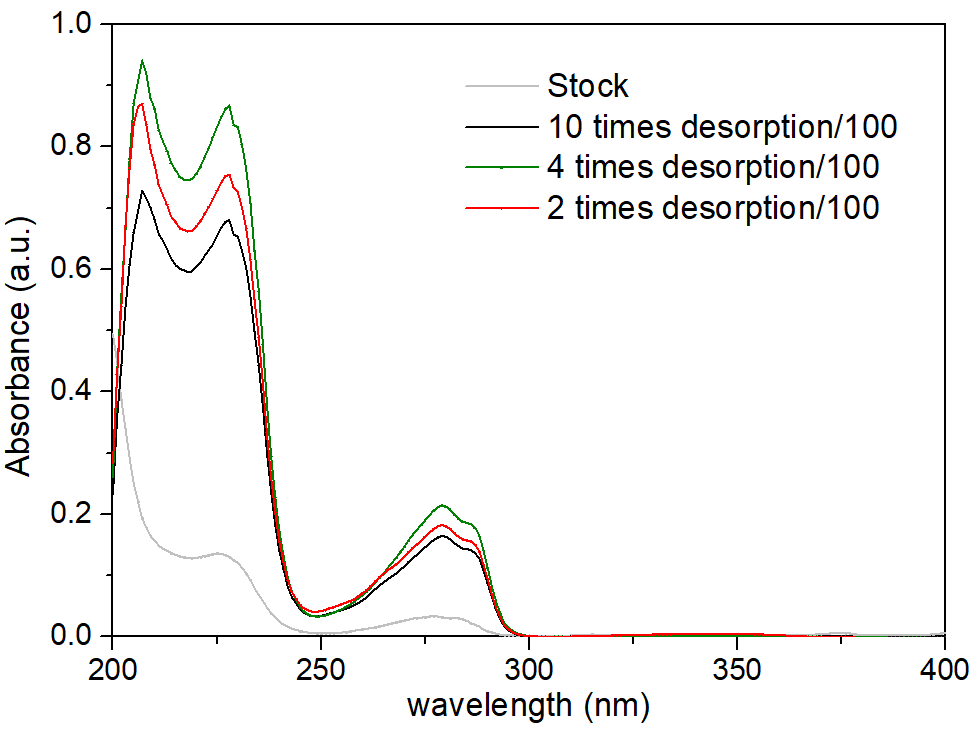

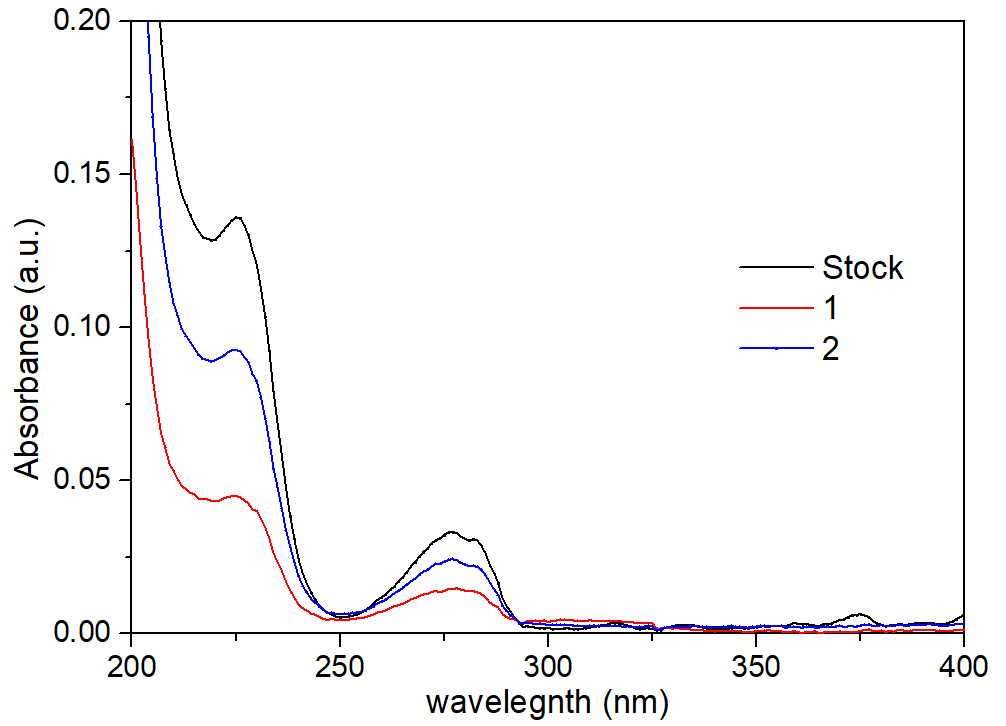

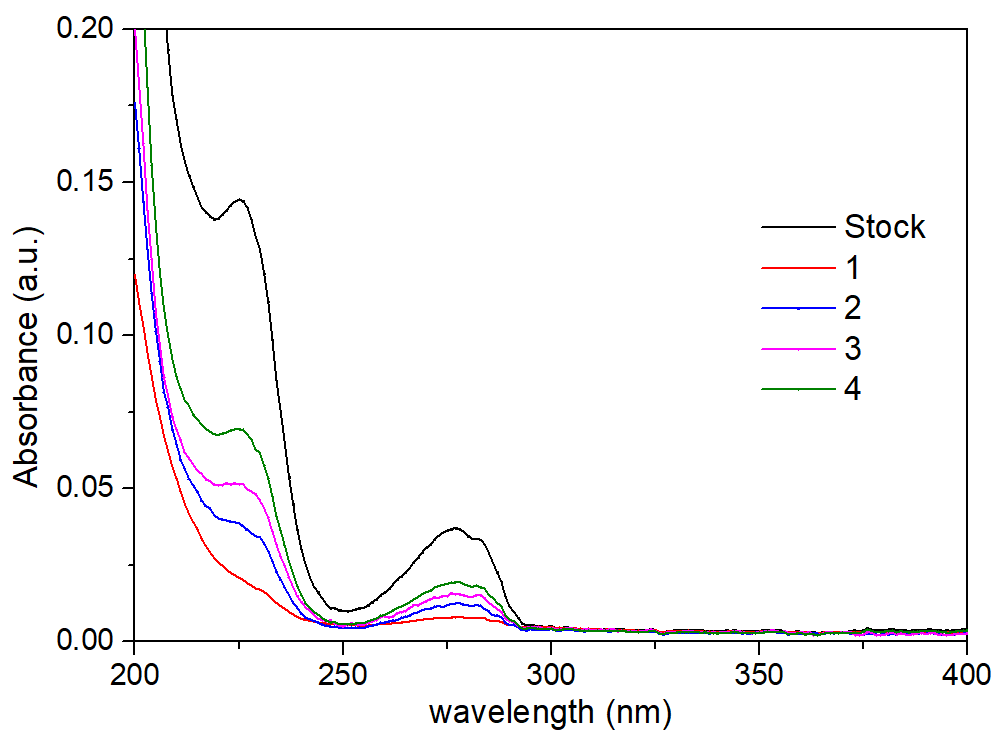

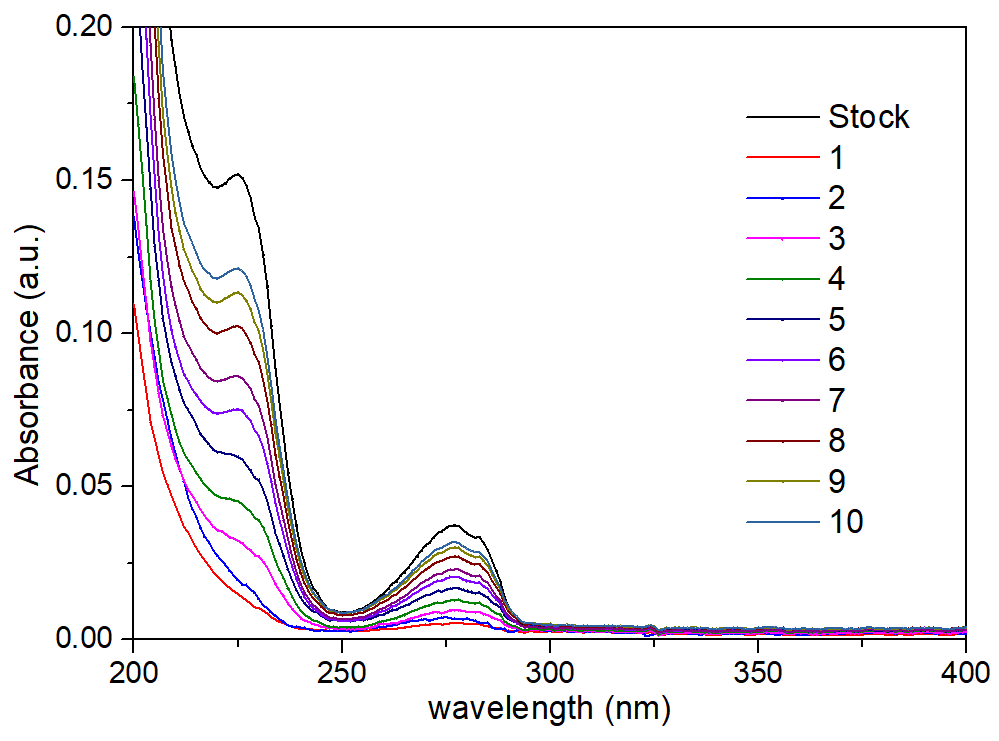


a

b

c

d

**Supplementary Figure 14 |** Adsorption and desorption of pollutants by MN-PCDP. UV–vis spectra recorded as a function of different cycles by MN-PCDP for removal BPA (0.01 mM) in different methods: **a** 100 mL for 10 times, **b** 250 mL for 4 times and **c** 500 mL for 2 times. **d** UV–vis spectra recorded desorption of pollutants by MN-PCDP in above three methods.

**Supplementary Table 4 |** The removal efficiency of BPA by different method in Figure S14

| Removal efficiency (%) | 100 mL for 10 times | 250 mL for  4 times | 500 mL for  2 times |
| --- | --- | --- | --- |
| 1 | 88.54 | 78.56 | 55.80 |
| 2 | 82.78 | 66.15 | 26.65 |
| 3 | 74.36 | 57.85 |  |
| 4 | 65.27 | 47.76 |  |
| 5 | 54.85 |  |  |
| 6 | 44.96 |  |  |
| 7 | 38.56 |  |  |
| 8 | 27.01 |  |  |
| 9 | 19.19 |  |  |
| 10 | 14.31 |  |  |
| Average | 50.98 | 62.58 | 41.22 |


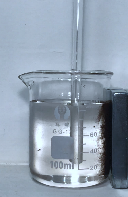

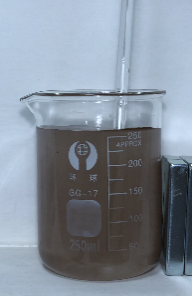


b


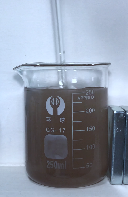


0 s

15 s


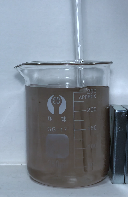

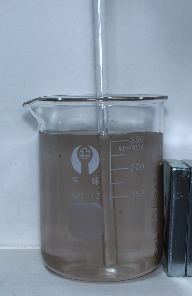

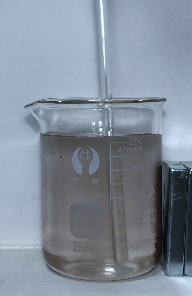

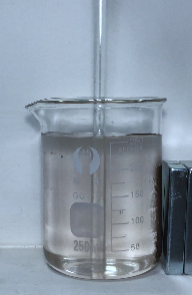

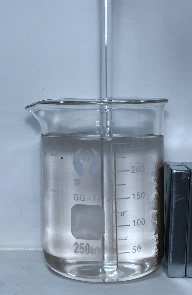

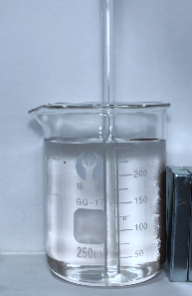


30 s

45 s

60 s

90 s

120 s

150 s min


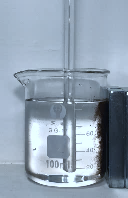

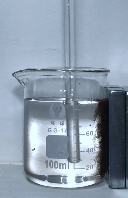

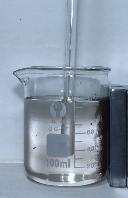

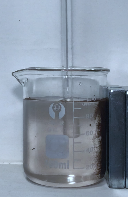

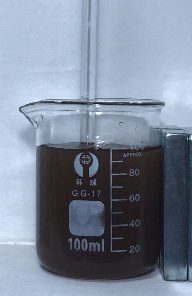

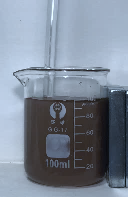

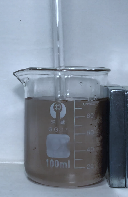


0 s

15 s

30 s

45 s

60 s

75 s

90 s

120 s min

a


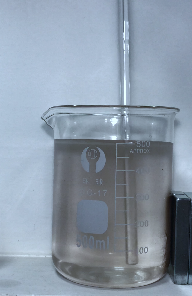

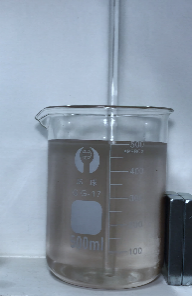

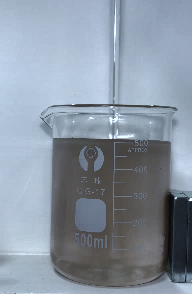

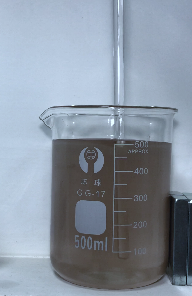

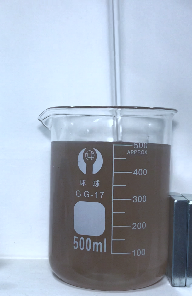


0 s

30 s

60 s

120 s min

90 s


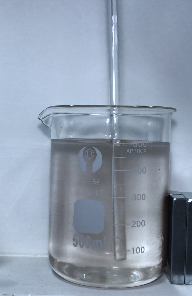


150 s min


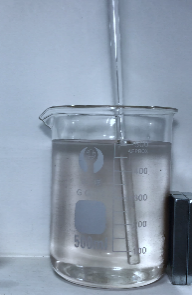


180 s min


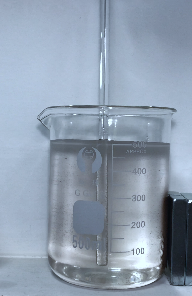


210 s min

c

**Supplementary Figure 15 |** Images constant times about magnetic separation of MN-PCDP in **a** 100 mL, **b** 250 mL and **c** 500 mL pollution solution.


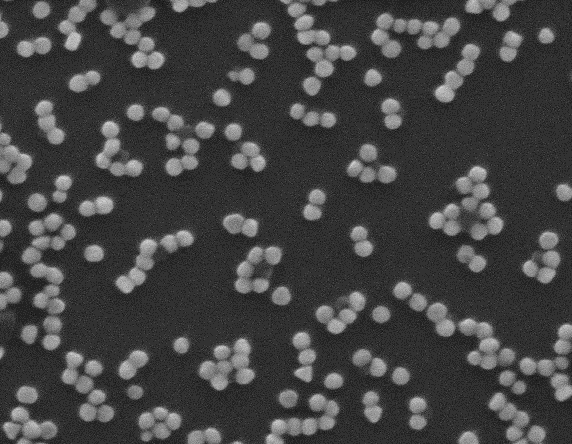


a

500 nm


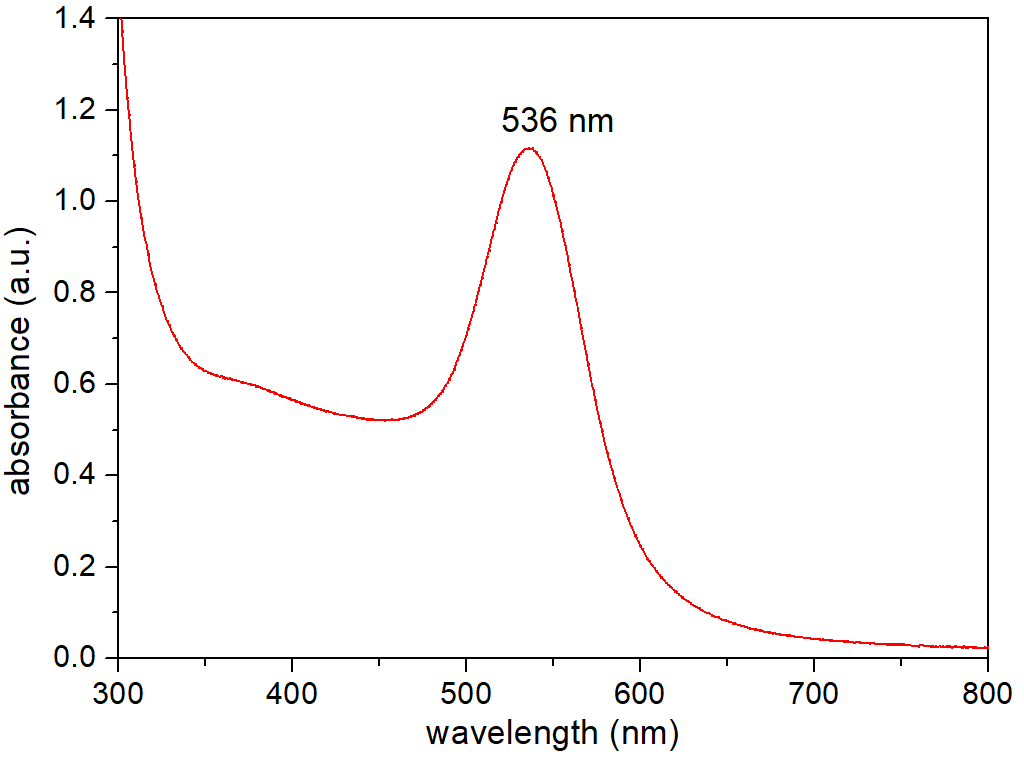


b

**Supplementary Figure 16 | a** SEM image and **b** UV-vis spectroscopy of Au NPs with ~55 nm in diameter.


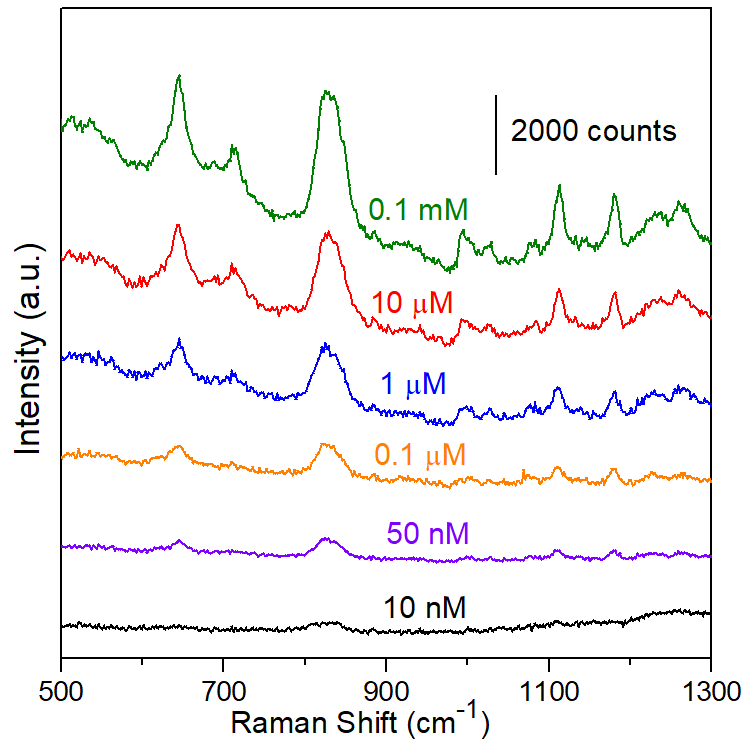

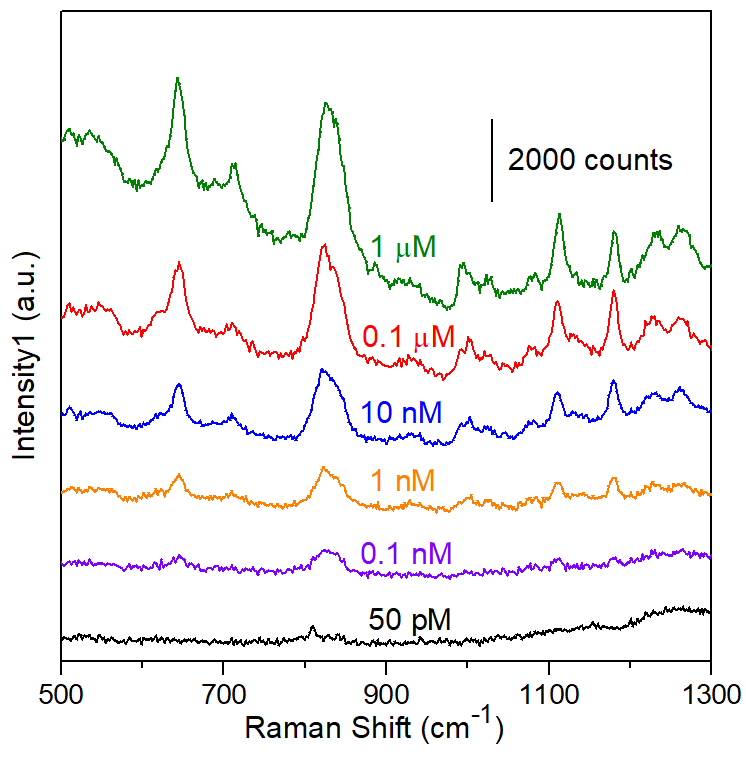


BPA

BPA enrichment

a

b

**Supplementary Figure 17 |** Enhanced Raman spectra of BPA **a** before and **b** after the enrichment of MN-PCDP adsorbent. The characteristic peaks of BPA were at 645, 830, 1108 and 1179 cm^-1^.


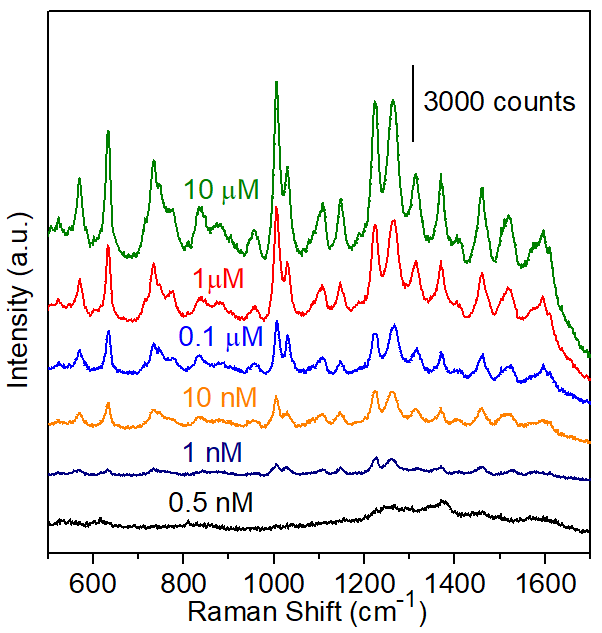


Carbendazim

a


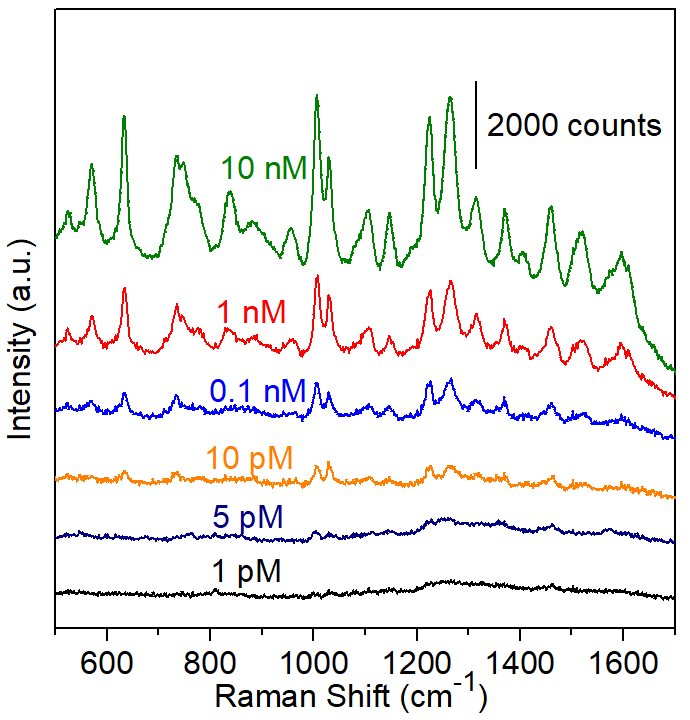


b

Carbendazim enrichment

**Supplementary Figure 18 |** Enhanced Raman spectra of carbendazim **a** before and **b** after the enrichment of MN-PCDP adsorbent. The characteristic peaks of carbendazim were at 632, 1004, 1222 and 1263 cm^-1^.


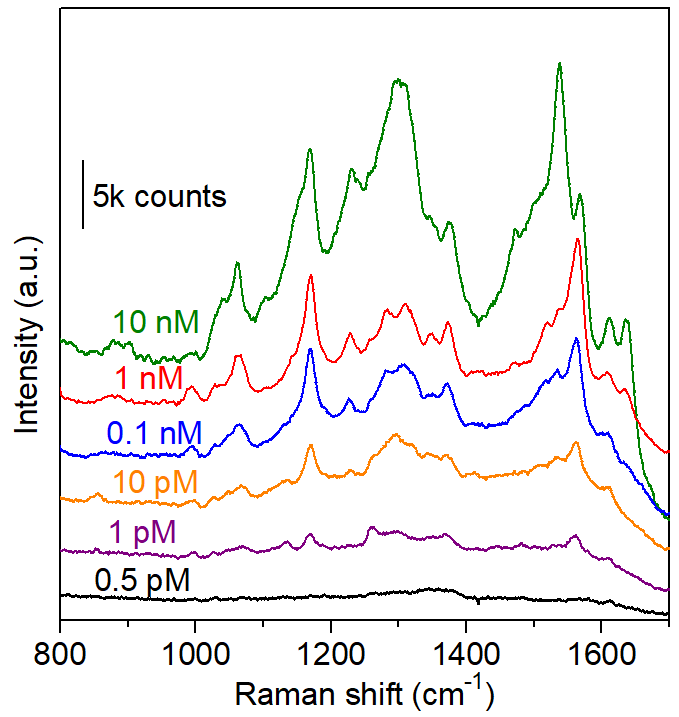

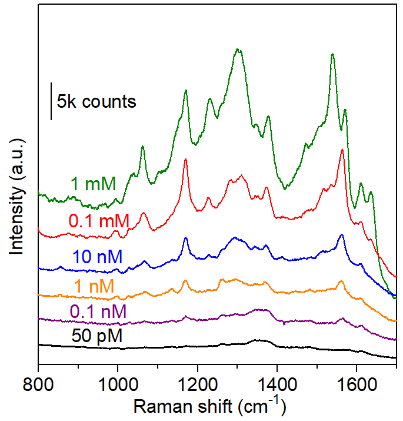


Diquat

a

Diquat enrichment

b

**Supplementary Figure 19** | Enhanced Raman spectra of diquat **a** before and **b** after the enrichment of MN-PCDP adsorbent. The characteristic peaks of diquat were at 1061, 1170, 1376 and 1563 cm^-1^.


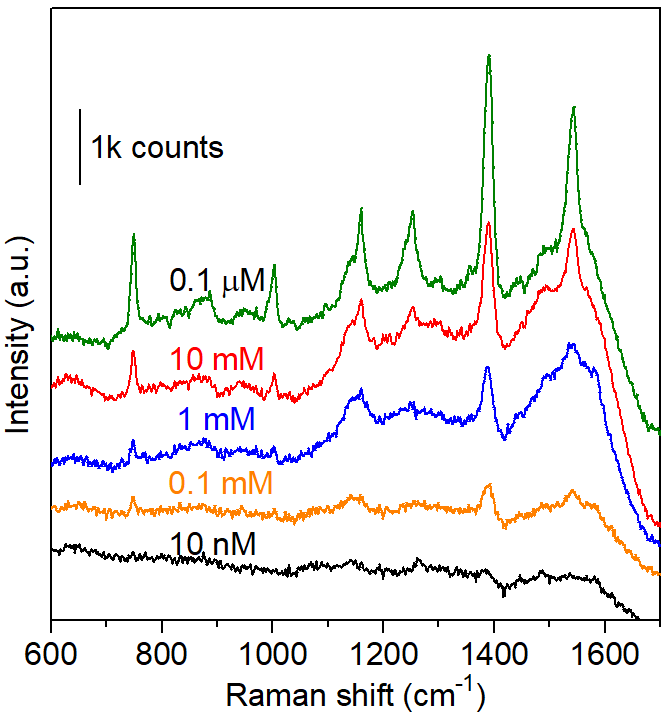

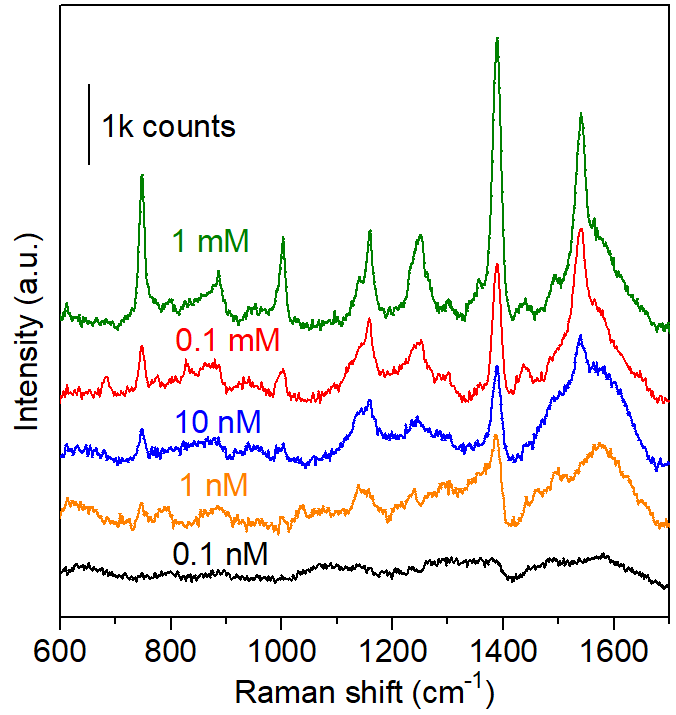


a

Anthracene

Anthracene

enrichment

b

**Supplementary Figure 20 |** Enhanced Raman spectra of anthracene **a** before and **b** after the enrichment of MN-PCDP adsorbent. The characteristic peaks of anthracene were at 748, 1160, 1389, and 1540 cm^-1^.


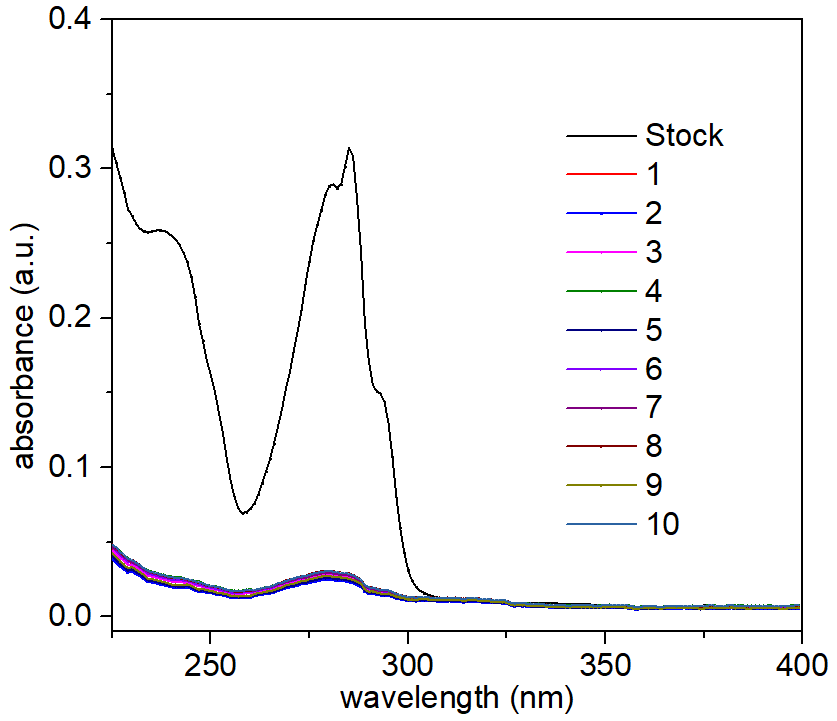

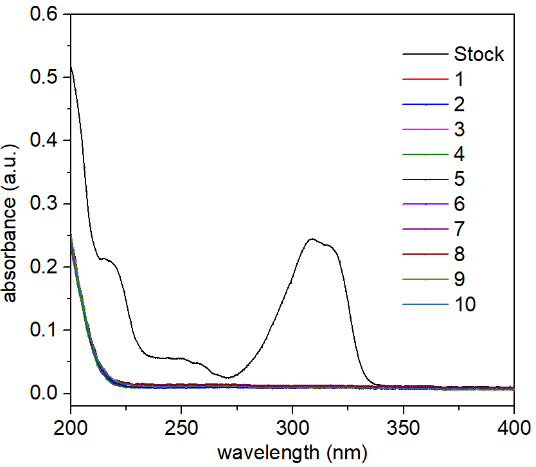


a

b


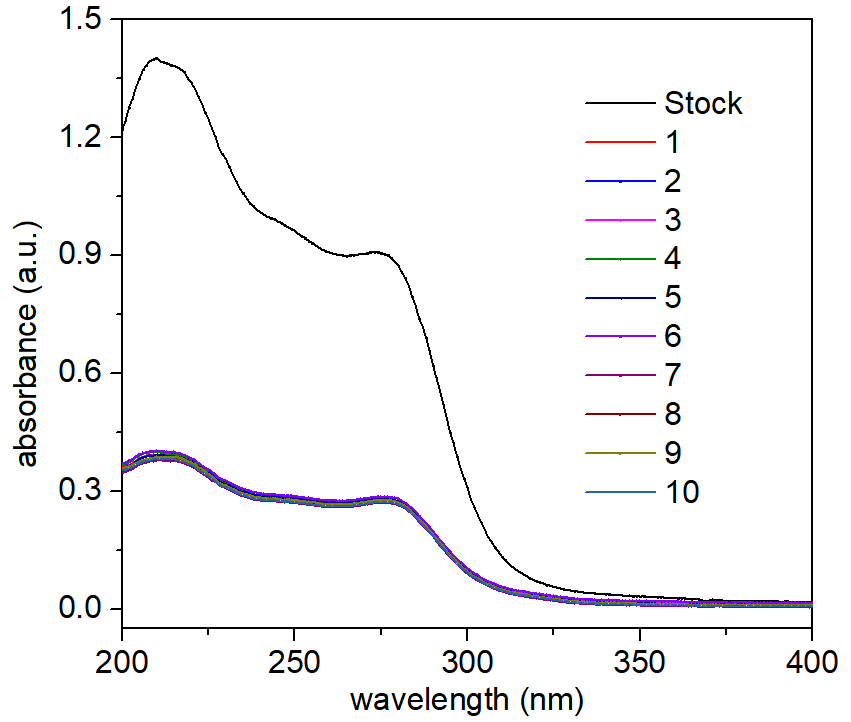


c

d

BPA


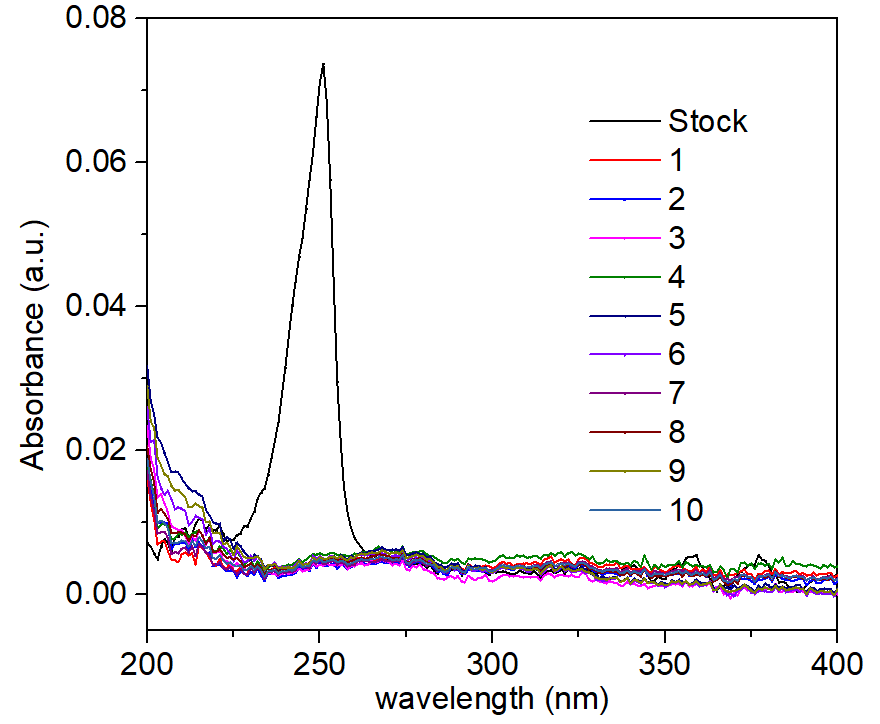


e

Carbendazim

TMTD

Diquat

Anthracene


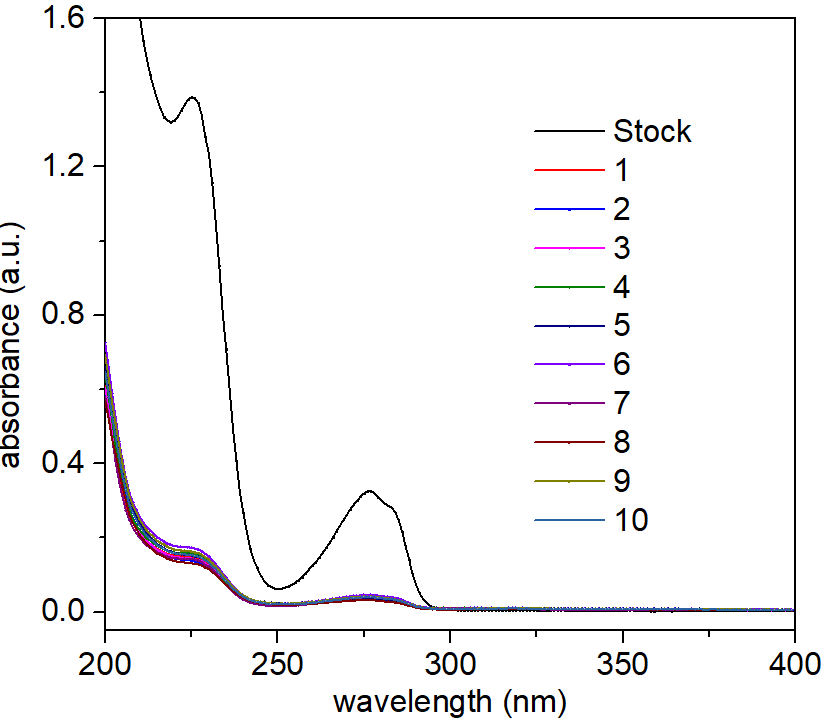


**Supplementary Figure 21** | Adsorption reproducibility of MN-PCDP. UV–vis spectra recorded as a function of different absorption experiments by MN-PCDP for removal BPA (0.1 mM, **a**), carbendazim (0.1 mM, **b**), TMTD (0.1 mM, **c**), diquat (0.01 mM, **d**) and anthracene (0.01 mM, **e**) by MN-PCDP (1 mg mL^-1^).

**Supplementary Table 5 |** The enrichment efficiency of BPA, carbendazim, TMTD, diquat and anthracene by different absorption cycles in Figure S21

| Removal efficiency (%) | BPA | Carbendazim | TMTD | Diquat | Anthracene |
| --- | --- | --- | --- | --- | --- |
| 1 | 89.17 | 91.37 | 69.20 | 96.17 | 93.24 |
| 2 | 90.01 | 92.67 | 68.98 | 96.05 | 94.59 |
| 3 | 89.59 | 91.62 | 69.29 | 96.03 | 94.59 |
| 4 | 88.63 | 90.94 | 69.17 | 96.42 | 93.24 |
| 5 | 89.01 | 90.94 | 69.15 | 96.37 | 93.24 |
| 6 | 87.51 | 92.31 | 68.42 | 94.92 | 93.24 |
| 7 | 89.71 | 91.20 | 70.15 | 94.70 | 94.59 |
| 8 | 90.51 | 91.21 | 69.74 | 95.06 | 93.24 |
| 9 | 88.26 | 90.78 | 69.41 | 96.08 | 93.24 |
| 10 | 88.93 | 91.92 | 69.92 | 95.89 | 94.59 |
| Average | 89.13 | 91.49 | 69.34 | 96.17 | 94.78 |
| RSD | 0.98% | 0.69% | 0.71% | 0.66% | 0.074% |


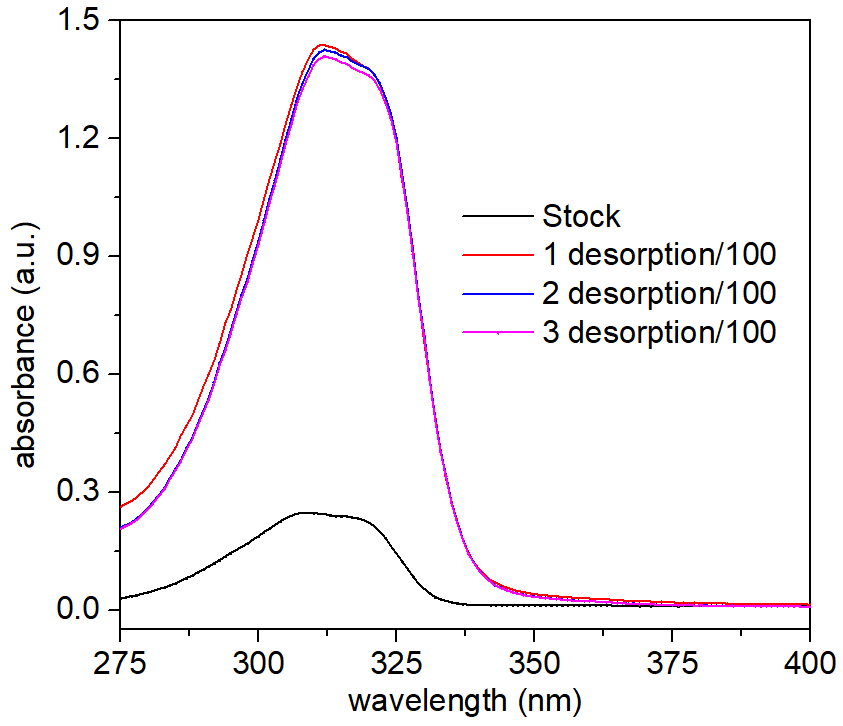

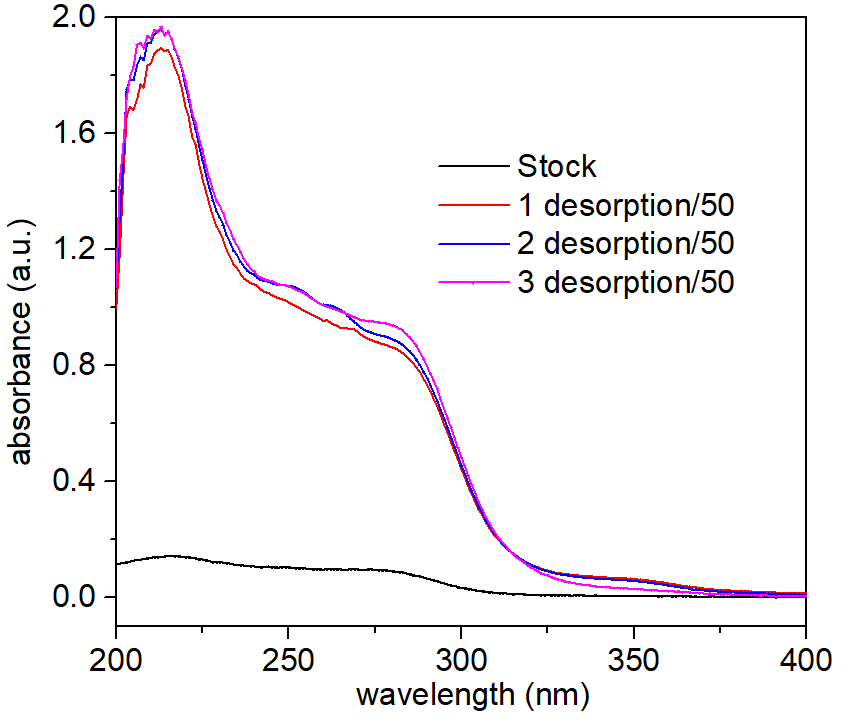


c


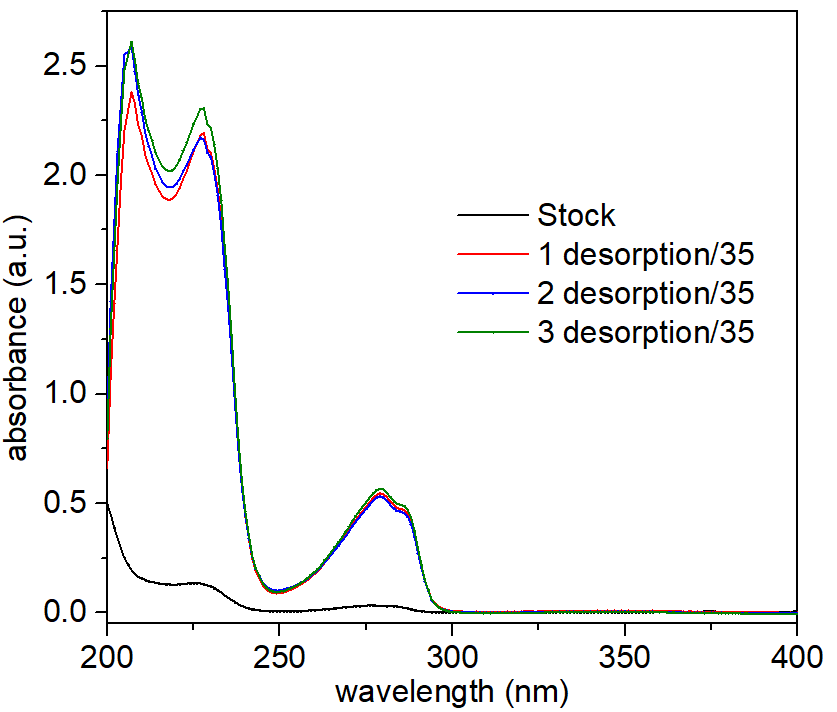


a


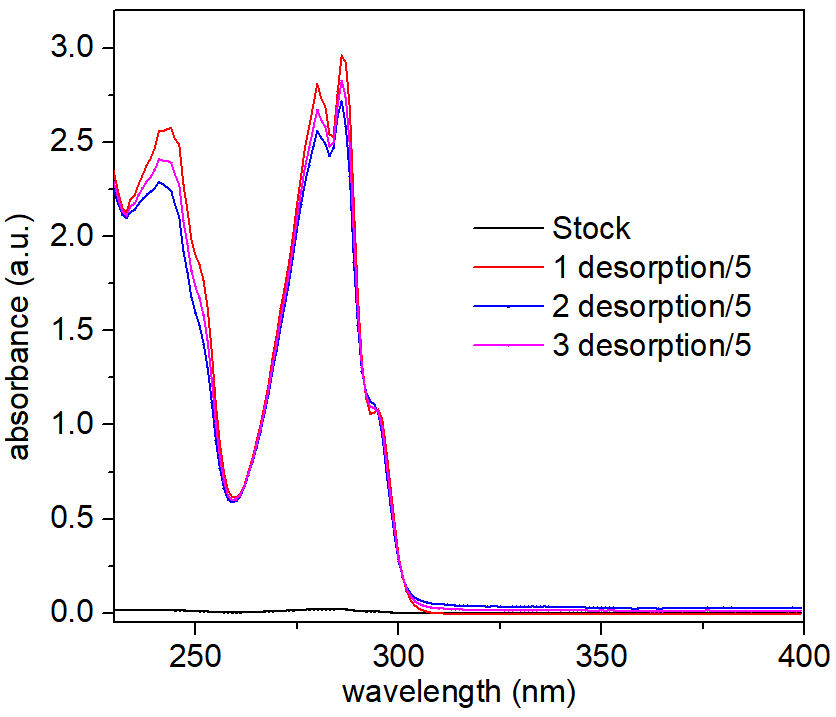


b

d

e

Anthracene

BPA

Carbendazim

TMTD

Diquat

**Supplementary Figure 22 |** Enrichment reproducibility of MN-PCDP. UV-vis spectra recorded as a function of triplicate desorption experiments by MN-PCDP for enrichment BPA (0.01 mM, **a**), carbendazim (0.01 mM, **b**), TMTD (0.01 mM, **c**), diquat (0.01 mM, **d**) and anthracene (0.01 mM, **e**) by MN-PCDP (100 mg in 250 mL×4 times absorption).

**Supplementary Table 6 |** The enrichment efficiency of BPA, carbendazim, TMTD, diquat and anthracene by different enrichment cycles in Figure S22

| Enrichment efficiency (times) | BPA | Carbendazim | TMTD | Diquat | Anthracene |
| --- | --- | --- | --- | --- | --- |
| 1 | 605.29 | 642.71 | 435.12 | 598.02 | 521.49 |
| 2 | 595.01 | 589.34 | 449.48 | 586.95 | 547.61 |
| 3 | 624.69 | 613.36 | 475.62 | 572.63 | 526.94 |
| Average | 608.33 | 615.14 | 453.41 | 585.87 | 532.01 |
| RSD | 2.48% | 4.34% | 4.53% | 2.17% | 2.59% |

**Supplementary Figure 23 |** Enrichment efficiencies of five organic pollutants after enrichment process of MN-PCDP. The data are reported as the average enrichment of triplicate experiments. Error bars mean standard deviations.

1 cm

a

0.2 mm

b

**Supplementary Figure 24 |** **a** Optical photograph and **b** bright-field optical images of analyte and Au nanoparticles on the hydrophobic slippery SERS platform.

Sample 1

a

Sample 2

b

Sample 3

c

d

Sample 4

Sample 5

e

**Supplementary Figure 25 |** Enhanced Raman spectra of TMTD (1 pM) in five independent batches after the enrichment of MN-PCDP measured by hydrophobic slippery SERS platform.

b

Sample 2

a

Sample 1

c

Sample 3

d

Sample 4

e

Sample 5

**Supplementary Figure 26 |** Enhanced Raman spectra of TMTD (0.5 pM) in five independent batches after the enrichment of MN-PCDP measured by hydrophobic slippery SERS platform.

Sample 2

b

Sample 1

a

Sample 4

d

Sample 3

c

Sample 5

e

**Supplementary Figure 27 |** Enhanced Raman spectra of TMTD (0.1 pM) in five independent batches after the enrichment of MN-PCDP measured by hydrophobic slippery SERS platform.

a

Sample 1

b

Sample 2

d

Sample 4

e

Sample 5

Sample 3

c

**Supplementary Figure 28 |** Enhanced Raman spectra of TMTD (50 fM) in five independent batches after the enrichment of MN-PCDP measured by hydrophobic slippery SERS platform.

a

b

Sample 1

c

Sample 2

Sample 3

e

Sample 5

d

Sample 4

**Supplementary Figure 29 |** Enhanced Raman spectra of TMTD (10 fM) in five independent batches after the enrichment of MN-PCDP measured by hydrophobic slippery SERS platform.

Sample 1

a

Sample 2

b

Sample 3

c

Sample 4

e

Sample 5

e

**Supplementary Figure 30 |** Enhanced Raman spectra of TMTD (5 fM) in five independent batches after the enrichment of MN-PCDP measured by hydrophobic slippery SERS platform.

a

b

**Supplementary Figure 31 |** Enhanced Raman spectra of TMTD **a** before and **b** after the enrichment of MN-PCDP adsorbent measured by aggregating approach with Au colloid. The characteristic peaks of TMTD were at 558 and 1379 cm^-1^.

a

1 nM

0.1 nM

10 pM

1 pM

c

b

d

**Supplementary Figure 32 |** Enhanced Raman spectra of TMTD with **a** 1 nM, **b** 0.1nM, **c** 10 pM and **d** 1pM in five independent batches after the enrichment of MN-PCDP adsorbent measured by aggregating approach with Au colloid.

a

b

**Supplementary Figure 33** | Fluorescence spectra of BPA **a** before and **b** after the enrichment of MN-PCDP adsorbent. The concentration in the parentheses was estimated the concentration after enrichment (enrichment ~500 fold).

SY

Mix

MG

a

b

c

desorption

absorption

**Supplementary Figure 34** | Separation of SY/MG mixture dyes: **a** photograph of SY (0.01 mM), MG (0.01 mM) and SY/MG (both with 0.01 mM) mixture solution, **b** photograph of SY/MG mixture solution after absorption by MN-PCDP, **c** photograph of SY/MG mixture solution after desorption by MN-PCDP.

**Supplementary Figure 35** | Separation of CCC/TMTD mixture pesticides: UV−vis spectra of CCC (1 mM), TMTD (0.1 mM) and CCC (1 mM)/TMTD (0.1 mM) mixture solution before and after absorption, desorption.

a

b

**Supplementary Figure 36** | UV−vis spectra about selective extraction and enrichment of organic pollutant molecules (0.01 mM) from practical samples: **a** BPA in industrial wastewater, **b** MG in pond water.

a

b

**Supplementary Figure 37** | Raman spectra about selective extraction and enrichment of organic pollutant molecules (0.1 µM) from practical samples: **a** BPA in industrial wastewater, **b** MG in pond water.

**Supplementary Figure 38 |** The average removal efficiency of BPA by MN-PCDP after consecutive regeneration cycles. MN-PCDP was regenerated by washing with ethanol at room temperature. The data are reported as the average of triplicate experiments with adsorption time of 1 min and washing time of 10 min. The data are reported as the average uptake of triplicate experiments. Error bars mean standard deviations.

1.15 µm

Fe_3_O_4_

Au

~2 µm

1 µm

a

~10 µm

Fe_3_O_4_

Au

b

**Supplementary Figure 39** | Schematic of measurements at single nanoparticle level. by MN-PCDP. **a** Single Fe_3_O_4_-Au core-shell structure nanoparticle with crosslinked porous β-CD polymer on the surface, detection solution with target molecular is concentrated from 50 µL to 3.4×10^-8^ µL (enrichment ~1.5×10^9^ fold). **b** MN-PCDP with Au NPs on the surface, detection solution with target molecular is concentrated from 50 µL to 4.2×10^-6^ µL (enrichment ~1.2×10^7^ fold).
